# Broad Chain-Length Specificity of the Alkane-Forming Enzymes NoCER1A and NoCER3A/B in *Nymphaea odorata*

**DOI:** 10.1093/pcp/pcad168

**Published:** 2024-02-09

**Authors:** Hisae Kojima, Kanta Yamamoto, Takamasa Suzuki, Yuri Hayakawa, Tomoko Niwa, Kenro Tokuhiro, Satoshi Katahira, Tetsuya Higashiyama, Sumie Ishiguro

**Affiliations:** Technical Center, Nagoya University, Nagoya, 464-8601 Japan; Graduate School of Bioagricultural Sciences, Nagoya University, Nagoya, 464-8601 Japan; Graduate School of Bioagricultural Sciences, Nagoya University, Nagoya, 464-8601 Japan; College of Bioscience and Biotechnology, Chubu University, Kasugai, 487-8501 Japan; Graduate School of Bioagricultural Sciences, Nagoya University, Nagoya, 464-8601 Japan; Graduate School of Bioagricultural Sciences, Nagoya University, Nagoya, 464-8601 Japan; Toyota Central R&D Labs., Inc., Nagakute, 480-1192 Japan; Toyota Central R&D Labs., Inc., Nagakute, 480-1192 Japan; Institute of Transformative Bio-Molecules (WPI-ITbM), Nagoya University, Nagoya, 464-8601 Japan; Graduate School of Science, The University of Tokyo, Tokyo, 113-0033 Japan; Graduate School of Bioagricultural Sciences, Nagoya University, Nagoya, 464-8601 Japan

**Keywords:** Alkane-forming enzyme, *Arabidopsis thaliana*, Carbon chain length, *Nymphaea odorata*, Tobacco BY-2 cell line, Wax crystals

## Abstract

Many terrestrial plants produce large quantities of alkanes for use in epicuticular wax and the pollen coat. However, their carbon chains must be long to be useful as fuel or as a petrochemical feedstock. Here, we focus on *Nymphaea odorata*, which produces relatively short alkanes in its anthers. We identified orthologs of the Arabidopsis alkane biosynthesis genes *AtCER1* and *AtCER3* in *N. odorata* and designated them *NoCER1A, NoCER3A* and *NoCER3B*. Expression analysis of *NoCER1A* and *NoCER3A/B* in Arabidopsis *cer* mutants revealed that the *N. odorata* enzymes cooperated with the Arabidopsis enzymes and that the NoCER1A produced shorter alkanes than AtCER1, regardless of which CER3 protein it interacted with. These results indicate that AtCER1 frequently uses a C30 substrate, whereas NoCER1A, NoCER3A/B and AtCER3 react with a broad range of substrate chain lengths. The incorporation of shorter alkanes disturbed the formation of wax crystals required for water-repellent activity in stems, suggesting that chain-length specificity is important for surface cleaning. Moreover, cultured tobacco cells expressing *NoCER1A* and *NoCER3A/B* effectively produced C19–C23 alkanes, indicating that the introduction of the two enzymes is sufficient to produce alkanes. Taken together, our findings suggest that these *N. odorata* enzymes may be useful for the biological production of alkanes of specific lengths. 3D modeling revealed that CER1s and CER3s share a similar structure that consists of N- and C-terminal domains, in which their predicted active sites are respectively located. We predicted the complex structure of both enzymes and found a cavity that connects their active sites.

## Introduction

Many land plants develop cuticles on the surface of their aboveground parts to protect the plants from environmental stresses including desiccation, rainfall, UV irradiation and pathogen attack. The cuticle consists of two types of lipidic molecule: cutin and cuticular waxes. Cutin is a polyester mainly comprising hydroxy and epoxy 16 and 18 carbon (C16 and C18) fatty acids and glycerol, whereas cuticular waxes are mixtures of alkanes and other aliphatic compounds derived from very-long-chain (VLC, i.e. C21 and longer carbon chain) fatty acids (Bernard and Joubès 2013, Lee and Suh 2015, Fich et al. 2016). The latter are classified into intracuticular waxes, which are localized in the cutin layer, and epicuticular waxes, which form the outer coat of the cuticle (Preuss et al. 1993, Aarts et al. 1995, Hülskamp et al. 1995, Fiebig et al. 2000, Ariizumi et al. 2003, Chen et al. 2003, Ishiguro et al. 2010, Haslam et al. 2015). Epicuticular waxes often form small crystals that enhance the water repellency of plant surfaces (Neinhuis et al. 1992). Alkanes are also major constituents of the pollen coat, the outermost surface structure of pollen grains that is required for adhesion and interrecognition between pollen grains and stigmas (Preuss et al. 1993, Hülskamp et al. 1995).

In Arabidopsis (*Arabidopsis thaliana*), the stem cuticular wax contains a mixture of alkanes and related molecules including aldehydes, alcohols, ketones and fatty acids ranging from C26 to C31 in length (Jenks et al. 1995). The biosynthetic enzymes responsible for synthesizing these molecules are encoded by a subset of *ECERIFERUM* (*CER*) genes, and the mutations in these genes cause wax-deficient phenotypes on the stem surface (Koornneef et al. 1989). To date, >25 *CER* and related genes in the Arabidopsis genome are thought to be involved in wax biosynthesis, transport or regulation (Bernard and Joubès 2013, Lee and Suh 2015).

Alkane synthesis is initiated by the activation of C16 and C18 fatty acids to form acyl-coenzyme A (CoA) by a long-chain acyl-CoA synthetase (lacs) in the endoplasmic reticulum (ER), where the following process occurs. *CER8*/*LACS1* and related genes are thought to be involved in this process (Lü et al. 2009). *CER6*, also known as *CUT1*, encodes a β-ketoacyl-CoA synthase, a key component of the elongation system (Millar et al. 1999, Fiebig et al. 2000). Mutations in this gene cause the reduction of all wax monomers longer than C24, indicating that CER6 is required for the elongation of fatty acyl-CoA beyond C24 (Millar et al. 1999). CER6 alone can generate molecules up to C28 in length, but the co-expression of CER2 or its related proteins with CER6 can facilitate the production of even longer fatty acids (i.e. up to C34) (Haslam et al. 2012, 2015). In rice, a similar interaction has been reported between WSL4 (a CER6 homolog) and OsCER2 (Wang et al. 2017). After elongation, the VLC acyl-CoA enters either the alkane-forming or alcohol-forming pathway. In the alkane-forming pathway, the VLC acyl-CoA is reduced to an aldehyde by *CER3* (also known as *FLP, WAX2* or *YRE*)-encoded fatty acyl-CoA reductase, and this aldehyde is then converted to an alkane that is one carbon shorter in chain length via a *CER1*-encoded aldehyde decarbonylase (Aarts et al. 1995, Ariizumi et al. 2003, Chen et al. 2003, Kurata et al. 2003, Rowland et al. 2007, Bourdenx et al. 2011, Bernard et al. 2012). A similar two-step alkane-forming system also exists in cyanobacteria and insects, though the activity of the second enzyme is different. The byproduct of the CER1 reaction is carbon monoxide, which is a characteristic of plant alkane-forming enzymes. In contrast, cyanobacteria and animal enzymes generate formic acid and carbon dioxide, respectively (Cheesbrough and Kolattukudy 1984, Warui et al. 2011, Qiu et al. 2012).

CER3 and CER1 are localized in the ER membrane and are thought to form a complex *in vivo* (Aarts et al. 1995, Millar et al. 1999, Bernard et al. 2012). Although their enzyme activities are different, they are similar in amino acid sequence and probably originated in the common ancestor of the Viridiplantae (Wang et al. 2019, Chaudhary et al. 2021). CER1 is presumably a non-heme iron enzyme that possesses a tripartite His cluster that is essential for its enzyme activity, whereas this motif is not necessary for functional CER3 (Aarts et al. 1995, Chen et al. 2003, Bernard et al. 2012). Another conserved but functionally unknown WAX2 domain is in their C-terminal regions. Bernard et al. (2012) showed that the co-expression of CER1 and CER3 in a manipulated yeast strain producing VLC acyl-CoA resulted in the production of C27–C31 alkanes, which matched the chain-length distribution of alkanes in CER1-overexpressing Arabidopsis plants (Bourdenx et al. 2011). This finding indicated that CER1 and CER3 constitute a core complex of alkane synthesis enzymes that show strict carbon chain-length specificity for C28 and longer substrates. In contrast, CER1-LIKE1, a homolog of CER1 that is predominantly expressed in flowers and siliques, involves the production of C25 and C27 alkanes (Greer et al. 2007, Pascal et al. 2019). It has been proposed that CER1 and CER1-LIKE1 form distinct complexes with CER3 that work in a complementary manner in wax synthesis in flowers. In rice, three CER1 homologs (OsGL1-4/OsCER1, OsGL1-5/WDA1 and OsGL1-6) and three CER3 homologs (OsGL1-1/WSL2, OsGL1-2 and OsGL1-3) coordinate during alkane production (Jung et al. 2006, Islam et al. 2009, Qin et al. 2011, Mao et al. 2012, Zhou et al. 2013, 2015, Ni et al. 2018).

In some plant families, including the Brassicaceae, secondary alcohols and ketones are generated by midchain alkane hydroxylase, which facilitates hydroxylation and oxidation at the central carbon of alkanes (Greer et al. 2007). Most alkanes, secondary alcohols and ketones generated by this pathway are odd-numbered carbon molecules, since the terminal carbon atoms of the VLC acyl precursors, which usually have an even carbon number, are removed during the CER1-catalyzed decarbonylation step. In the alcohol-forming pathway, the VLC acyl-CoA is presumed to be converted to a potential intermediate aldehyde then changed to a primary alcohol by a single reducing enzyme, CER4 (Rowland et al. 2007). This alcohol is subsequently used for ester formation, predominantly with C16 fatty acids (Lai et al. 2007).

Since alkanes are major components of fuels used by internal-combustion engines, efforts have been made to develop biological production of alkanes by microorganisms. For example, metabolically modified *Escherichia coli* harboring the acyl–acyl carrier protein reductases and aldehyde-deformylating oxygenases from cyanobacteria have been demonstrated to produce C13–C17 alkanes and alkenes, which are appropriate for diesel fuel (Schirmer et al. 2010). Expression of *Clostridium acetobutylicum* fatty acyl-CoA reductase and Arabidopsis CER1 with other enzymes in *E. coli* resulted in the production of relatively short C9–C14 alkanes and alkenes (Choi and Lee 2013). Similar approaches have been developed by eukaryotic organisms such as yeast and algae using enzymes derived from various organisms. However, neither CER1 nor CER3 have yet been successfully used in any of these systems (Foo et al. 2017, Kang et al. 2017, Yunus et al. 2018, Hu et al. 2019, Li et al. 2020, Monteiro et al. 2022). One possible reason for their omission may be the specificity of Arabidopsis proteins for C28 and longer substrates (Bernard et al. 2012).

The epidermal cells of land plants preferentially produce nonvolatile VLC alkanes, which are used as structural components and surface protectants. However, some plants also produce volatile alkanes as odors in pollen and flowers (Mukherjee et al. 2013, Yuan et al. 2014, Malik and Barik 2016). These volatile alkanes are relatively short and are suitable for biofuels. Moreover, it is expected that plants may have alkane synthetic enzymes that are even more suitable for biofuel production than those found in yeast and algae, since in plants these enzymes are localized to the endoplasmic reticulum, the central place of lipid metabolism in eukaryotic cells (Henry et al. 2012, Garay et al. 2014). To determine whether this is the case, we have analyzed the pollen coat lipid composition of 290 plant species and found that the pollen coat of a water lily (*N. odorata*) contained shorter-chain alkanes (i.e. alkanes as small as C15). In this paper, we identified and characterized the CER1 and CER3 homologs of *N. odorata*, and found that they are able to produce shorter VLC alkanes in Arabidopsis plants and cultured tobacco cells.

## Result

### *Nymphaea odorata* Accumulates Shorter VLC Alkanes in its Pollen Coat than Arabidopsis

**Fig. 1:**
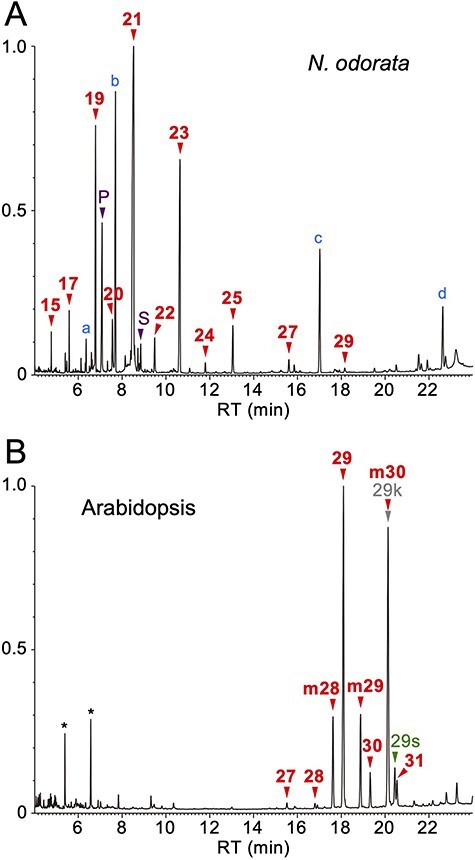
Total ion current chromatogram of the GC-MS analysis of pollen coat lipids. **(A)**  *Nymphaea odorata*. **(B)** Arabidopsis. Arrowheads indicate the peaks of aliphatic compounds of the indicated carbon chain length. Red, alkanes (numbers only); purple, fatty acids (P, palmitic acid; S, stearic acid); gray, ketones (labeled by “k”); green, secondary alcohols (labeled by “s”). Alkanes labeled by “m” are branched 2-methylalkanes. The peaks of 2-methyltriacontane and nonacosan-15-one are overlapping. The a–d in panel (A) are terpenoids (a, phytol; b, geranyllinalool; c, squalene; d, β-sitosterol). Asterisks in (B) are artifacts due to column contamination. RT, retention time. The vertical axis shows the relative intensity when the maximum peak is set as 1.0.

The pollen coat was first extracted from the pollen grains harvested from the fully opened flowers of a temperate water lily, *N. odorata*. Then the sample was analyzed using a gas chromatograph-mass spectrometer (GC-MS) equipped with a DB-1 nonpolar capillary column. This result showed that the *N. odorata* pollen coat included normal alkanes with carbon chain lengths ranging from C15 to C29, of which the C19, C21 and C23 molecules were the most abundant (**Fig. 1A**). Small amounts of C16 and C18 saturated fatty acids and some terpenoids were also detected. In Arabidopsis (*Arabidopsis thaliana* accession Columbia-0 (Col-0)), the pollen coat mainly consisted of normal and branched alkanes (Busta and Jetter 2017), of which the normal C29 alkane was the most abundant (**Fig. 1B**). Moreover, we also detected 15-nonacosanol and nonacosan-15-one, which are oxidized derivatives of normal C29 alkanes (Greer et al. 2007). The carbon chain lengths of Arabidopsis pollen coat components were distributed from C27 to C31, which is the most common range for angiosperms. Taken together, these results indicate that *N. odorata* can synthesize relatively short-chain alkanes for pollen coat production.

### Identification and Coding Sequence Cloning of *CER1* and *CER3* Homologs in *N. odorata*

Since the chain-length range of pollen coat alkanes depends on the characteristics of alkane synthetic enzymes, we expect that the enzymes present in *N. odorata* can produce shorter-chain alkanes. To test this possibility, we attempted to identify the homologs of Arabidopsis *CER1* (hereafter *AtCER1*) and *AtCER3* genes in *N. odorata*. An RNA sample isolated from young *N. odorata* anthers was reverse-transcribed and analyzed by paired-end tag sequencing on a next-generation sequencing platform. A BLAST search with *AtCER1* as the query sequence identified a sequence contig, named *CER1* homolog A, which matched the full-length protein-coding sequence (CDS) of *AtCER1*. Similarly, we also identified two contigs homologous to *AtCER3* CDS throughout their entire sequence (*CER3* homologs A and B). All three contigs contained SNPs that accounted for 2% of the total CDS nucleotides. Of these SNPs, one-third to one-half were nonsynonymous, and are presumably attributed to the multiploidy of this species (**Supplementary Table S1**) (Pellicer et al. 2013).

**Fig. 2:**
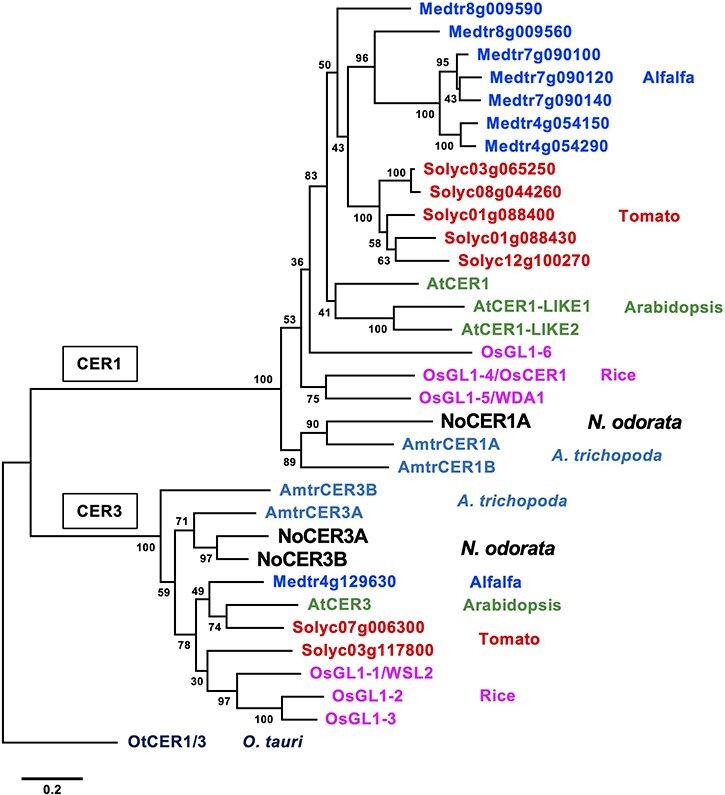
Structural relationships among the CER1 and CER3 proteins in *N. odorata* and representative angiosperms. Maximum likelihood phylogenetic tree of NoCER1A, NoCER3A, NoCER3B and their homologous proteins in tomato, alfalfa, Arabidopsis, rice and *A. trichopoda*. A predicted common homolog in *O. tauri* is added as an outgroup. Proteins are color coded according to species. Bootstrap support values from 1000 replicates are indicated. The bar represents 0.2 substitutions per site.

We prepared PCR primers for amplifying the full-length CDS of each of these genes. We obtained three CDS clones, designated *NoCER1A, NoCER3A* and *NoCER3B*. The entire amino-acid sequence of NoCER1A was compared to those of AtCER1 and its close homologs in alfalfa, tomato, rice and *Amborella trichopoda*, a species that, like water lily, belongs to the basal angiosperms (**Supplementary Fig. S1 and Supplementary Table S2**). Although the whole-gene sequences of the CER1 proteins were only somewhat similar, tripartite His clusters, which have been shown to be indispensable for the enzyme activity of AtCER1 (Chen et al. 2003, Bernard et al. 2012), were highly conserved in the N-terminal halves of the proteins sequences. Moreover, alignment of the whole NoCER3A and NoCER3B sequences with AtCER3 and its close homologs revealed that their C-terminal halves, including the WAX2 domain, were highly conserved (**Supplementary Fig. S2**). The NoCER1A and CER1 homologs also contained the WAX2 domain in their C-terminus, though this region showed less homology (**Supplementary Fig. S1**). We performed a phylogenetic analysis on all CER1 and CER3 homologs using whole-genome sequence data for each of these plant species as well as sequence data for a predicted common ancestral protein, OtCER1/3, from *Osterococcus tauri* (Chaudhary et al. 2021). Our data suggest that in CER1 homologs, the NoCER1A and *A. trichopoda* proteins first branched out from the others, which is consistent with the position of water lilies in angiosperm evolution (**Fig. 2**). Similarly, the NoCER3A, NoCER3B and *A. trichopoda* proteins also branched out earlier than others (**Fig. 2**). These results suggest that NoCER1 and NoCER3 belong to the CER1 and CER3 gene families, respectively, but they are structurally different from other angiosperm homologs. The sequence identities among CER1 homologs from different species (i.e. ∼50–60%) were relatively lower than those among CER3 homologs (i.e. ∼60–70%) (**Supplementary Fig. S3**). This pattern was particularly striking among CER1 homologs in *N. odorata, A. trichopoda*, Arabidopsis, and rice, which is consistent with the long-branch lengths found on the dendrogram (**Fig. 2**). Structural diversity was also observed among the intraspecific CER1 homologs in these species (**Supplementary Fig. S3**).

We also carried out RT-PCR using degenerate primers designed according to the sequences of 26 *CER1* and 15 *CER3* homologs found in public databases (**Supplementary Figs. S1 and S2 and Supplementary Table S3**). By use of DNA reverse transcribed from young anther RNA, we amplified a *CER1*-related sequence corresponding to *NoCER1A* and two *CER3*-related sequences that match *NoCER3A* and *NoCER3B*. This result supported that these genes are actively expressing *CER1* and *CER3* homologs in the anthers of *N. odorata*. In a recently published reference genome for tropical water lily, *Nymphaea colorata*, two *CER3* homologs corresponding to *NoCER3A* and *NoCER3B* and two tandem genes that were both similar to *NoCER1A* were identified (**Supplementary Fig. S4**) (Zhang et al. 2020). Hence, we concluded that we cloned the orthologs of *AtCER1* and *AtCER3* in *N. odorata*, and that they are actively transcribed in anthers.

### Co-expression of *NoCER1A* and *NoCER3A/B* Restored Alkane Biosynthesis in Arabidopsis *cer1 cer3* Double Mutants

In order to evaluate the enzyme activities of NoCER1A, NoCER3A and NoCER3B, we designed a complementation experiment using Arabidopsis *cer1 cer3* double mutants. The mutant alleles used in this study were *cer1-1* (SALK_008544) and *cer3-9* (GABI_177D09). Both are virtually null alleles disrupted by T-DNA insertions into the tenth exon and fourth intron of the respective genes (**Supplementary Figs. S5 and S6**) (Bourdenx et al. 2011, Xu et al. 2020). We connected the *NoCER1A* CDS with the *AtCER1* promoter (*ProAt1*; −1208 to −1; the A of the ATG start codon was set to +1). For CER3, we noted that the previous reports found that the exon 1 and intron 1 of *AtCER3* are important for efficient promoter activity (Kurata et al. 2003). Hence, we connected the CDS of *NoCER3A* and *NoCER3B* to an extended version of the *AtCER3* promoter (*ProAt3i*; −1748 to +144), which contained a 17-amino-acid N-terminal sequence of AtCER3 in exon 1 as well as all of intron 1 (**Supplementary Fig. S7**). The modified forms of NoCER3A and NoCER3B contained five and six amino-acid substitutions relative to the original NoCER3A and NoCER3B sequences, respectively (**Supplementary Fig. S7**). These genes were introduced into the double mutants by *Agrobacterium*-mediated transformation. Once grown, we extracted and analyzed the stem cuticular wax of the transgenic plants instead of pollen coat lipids, because the former is more abundant in Arabidopsis than the latter.

**Fig. 3:**
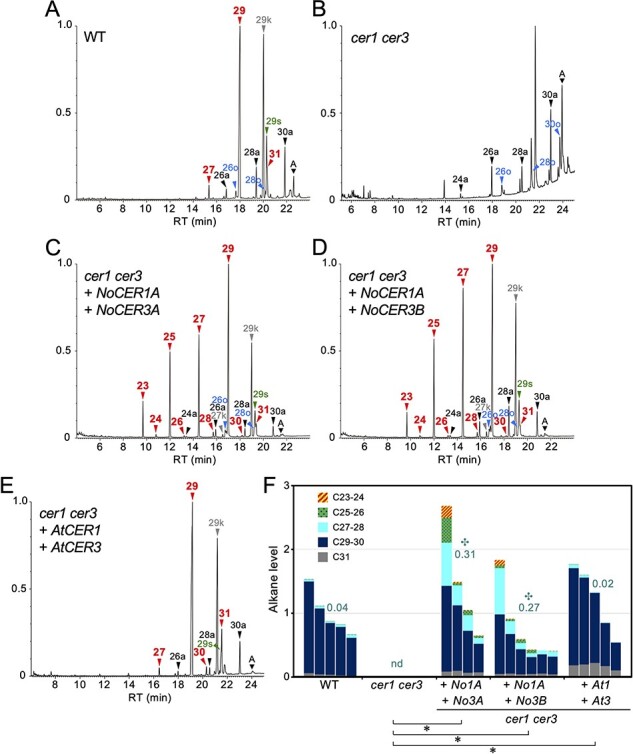
Restored alkane production in the stems of Arabidopsis *cer1 cer3* plants expressing both *NoCER1A* and *NoCER3A/B*. **(A–E)** Total ion current chromatogram of the GC-MS analysis. Typical results of the WT (A), *cer1 cer3* double mutants (B), *cer1 cer3* containing *ProAt1-NoCER1A* and *ProAt3i-NoCER3A* (C), *cer1 cer3* containing *ProAt1-NoCER1A* and *ProAt3i-NoCER3B* (D), and *cer1 cer3* containing *ProAt1-AtCER1* and *ProAt3i-AtCER3* (E) are shown. The vertical axis shows the relative intensity when the maximum peak is set as 1.0. Colored arrowheads indicate the peaks of compounds of the indicated carbon chain length. Red, alkanes (numbers only); black, aldehydes (labeled by ‘a’); gray, ketones (labeled by ‘k’); green, secondary alcohols (labeled by ‘s’); blue, primary alcohols (labeled by ‘o’). A, β-amyrin, an internal standard for quantification. Peaks with no labels are unidentified compounds. **(F)** Comparison of alkane levels shown as relative values compared to the average of the WT. Bars represent individual WT, *cer1 cer3* and independent T1 transformant plants. The ranges of carbon chain lengths are shown in different colors. Dark green values by the bars represent the average ratios of C28 and shorter alkanes against total alkanes. Asterisks indicate statistically significant differences of total alkane amount from *cer1 cer3* (✽) and of shorter alkane ratio from WT (✣), respectively (Steel’s test, *P *< 0.05). nd, not determined due to no detection of alkanes.

A GC-MS analysis of the wild-type Arabidopsis (WT) revealed that the stem cuticular wax contained a considerable amount of unbranched VLC alkanes of odd-number chain lengths (i.e. C27, C29 and C31); 15-nonacosanol (C29-15-alcohol) and 15-nonacosanone (C29-15-ketone), both of which are derived from C29 alkane; C26, C28 and C30 VLC aldehydes; and C26 and C28 primary alcohols (**Fig. 3A**). Of these products, C29 alkane and C29-15-ketone were the most abundant. This result was consistent with the previous reports (Jenks et al. 1996, Goodwin et al. 2005). Small amounts of the alkyl esters consisting of C12–C18 fatty acids and C24–C30 alcohols were detected by a GC-MS analysis performed with an increased sample amount and an extended retention time (**Supplementary Fig. S8**). In this experiment, minor components such as C24 and C30 alcohols, even-numbered alkanes, and odd-numbered aldehydes were also identified (**Supplementary Fig. S8**). In contrast, alkanes and the alkane derivatives were rarely detected on the surface of *cer1 cer3* stems, whereas small peaks of aldehydes and primary alcohols were readily observed (**Fig. 3B**).

Transgenic *cer1 cer3* plants in the T1 generation carrying *ProAt1-NoCER1A* and *ProAt3i-NoCER3A* genes produced a considerable amount of odd-numbered alkanes ranging C23–C29 in length, of which C29 was the most abundant (**Fig. 3C**). Similar results were obtained when *ProAt1-NoCER1A* and *ProAt3i-NoCER3B* were expressed (**Fig. 3D**). The alkane levels of these transformants were much higher than those of parental *cer1 cer3* double mutants and were comparable to those of WT plants (**Fig. 3F**). We used triterpenoid β-amyrin, a major constituent of intracuticular wax (Buschhaus and Jetter 2012), as an endogenous internal standard for alkane quantification instead of adding any exogenous internal standards, which might be included in the sample. To evaluate the enzyme preference for shorter molecules, we calculated the ratio of C28 and shorter alkanes against total alkane content. The values for *NoCER1A* and *NoCER3A*/*B* expressing plants reached ∼30%, whereas that of WT was less than 0.1% (**Fig. 3F**). As a control experiment, transgenic *cer1 cer3* plants containing *ProAt1-AtCER1* and *ProAt3i-AtCER3* genes produced stem wax in an amount and with a composition that was comparable to the WT (**Fig. 3E and F** and **Supplementary Fig. S7**). These results indicate that water lily enzymes had greater alkane synthetic activity and used broader chain-length acyl-CoA substrates relative to Arabidopsis enzymes.

To confirm these results, we used T2 transformants of the representative lines to determine the levels and carbon chain-length compositions of the major aliphatic components present in stem wax. We used six to eight T2 plants of each line and measured the quantities of the query compounds in a specified surface area. Measurements were normalized to an exogenously added C15 alkane standard. To estimate the amounts of aldehydes and alcohols, we determined 1.0 and 0.19 as relative response factors for these compound classes, respectively. As reported previously, *cer1* plants produced only trace levels of alkanes, indicating that CER1 activity is almost essential for alkane synthesis in stems (**Supplementary Fig. S9A**). This mutant accumulated C30 aldehyde approximately twice as much as the WT, although the levels of increased aldehydes were lower than the levels of decreased alkanes (**Supplementary Fig. S9B**). Moreover, alcohol production was also inhibited in the mutant (**Supplementary Fig. S9C**). These results suggest that the activity of CER3 was inhibited by the lack of CER1 protein or by the accumulation of the potentially toxic C30 aldehyde. In contrast, *cer3* mutant produced a low but still considerable amount of alkanes (**Supplementary Fig. S9A**). In this mutant, aldehydes and C26 and C28 alcohol production decreased, whereas C30 alcohol production increased (**Supplementary Fig. S9B and C**). Because the *cer3* allele used in this study was virtually a null allele, our results suggest that a fatty acid reductase other than CER3 contributed to C30 aldehyde formation, which was efficiently converted to C29 alkane and C30 alcohol. In *cer1 cer3* double mutants, the combined effects of both mutations were apparent. Alkanes and aldehyde levels were markedly decreased, whereas the abundance of C30 alcohol slightly increased, though its amount was much lower than the level of C29 alkane measured in the WT (**Supplementary Figs. S9A–C**). The alkane levels of T2 transformants co-expressing the *ProAt1-NoCER1A* and *ProAt3i-NoCER3A* (or *ProAt3i-NoCER3B*) genes in the *cer1 cer3* double-mutant background were lower than in parental T1 transformants, probably due to reductions in gene expression. Nevertheless, the preference of the enzymes for shorter substrates was clearly demonstrated; this is evident from the ratios of C28 and shorter alkanes to total alkanes in the transformants being higher than those of the WT (i.e. 0.24, 0.19 and 0.04 for NoCER1A + NoCER3A plants, NoCER1A + NoCER3B plants and WT plants, respectively) (**Supplementary Fig. S9A**). Aldehyde and alcohol levels were moderate in the transformants (**Supplementary Fig. S9B and C**).

### NoCER1 and NoCER3 Functionally Interact with Arabidopsis CER3 and CER1 Enzymes

**Fig. 4:**
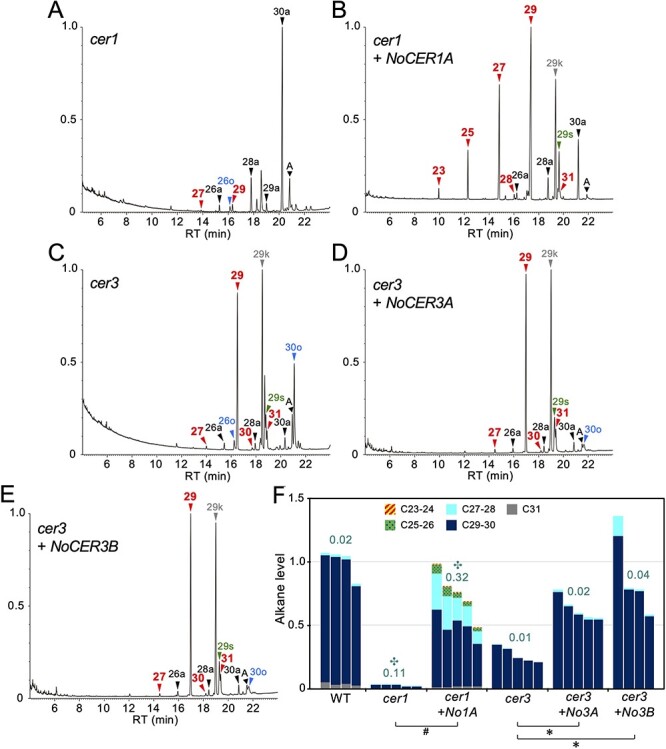
Contribution of NoCER1A and NoCER3A/B to the chain-length specificity of alkanes produced in Arabidopsis stems. **(A–E)** Total ion current chromatogram of GC-MS analysis. Typical results of *cer1* (A), *cer1* containing *ProAt1-NoCER1A* (B), *cer3* (C), *cer3* containing *ProAt1-NoCER3A* (D) and *cer3* containing *ProAt1-NoCER3B* (E) are shown. **(F)** Quantification of alkane levels. Statistically significant differences of shorter alkane ratio from WT (✣, Steel’s test, *P *< 0.05), of total alkane level from *cer1* (#, U-test, *P *< 0.05), and of total alkane levels from *cer3* (✽, Steel’s test, *P *< 0.05) are indicated. Other details are explained in **Fig. 3** legend.

Previous studies have shown that AtCER1 and AtCER3 interact with each other to form an enzymatic complex (Bernard et al. 2012). To examine whether *N. odorata* enzymes could work together with Arabidopsis enzymes, *NoCER1A* and *NoCER3A/B* genes were separately introduced into Arabidopsis *cer1* and *cer3* mutants, respectively, and their alkane synthetic activities were assayed. In this experiment, the *AtCER1* promoter was used for expressing the intact CDSs of both *NoCER1A* and *NoCER3A/B* genes (**Supplementary Fig. S7**). The expression of *NoCER1A* restored alkane synthesis activity in *cer1*, but the alkane chain length was shortened to C23, resembling the results of the *NoCER1*/*NoCER3* coexpression in the *cer1 cer3* double mutants (**Fig. 4B and F**). This indicated that NoCER1A had CER1 activity and worked together with AtCER3 (or subsidiary aldehyde-forming enzymes detected in the *cer3* mutant) to produce various alkane chain lengths. Transgenic *cer3* plants expressing *NoCER3A* or *NoCER3B* restored the levels of alkane production, and the lipid composition of their stem wax was similar to that of the WT (**Fig. 4D****–****F**). This indicated that NoCER3A/B had CER3 activity and worked together with AtCER1 but did not affect the chain length of the alkane product. In summary, our data showed that the CER1 and CER3 enzymes in *N. odorata* and Arabidopsis functionally interacted with each other, and CER1, but not CER3, determined chain-length specificity. In other words, NoCER1A combined with all examined CER3 generates various substrate chain lengths, whereas AtCER1 shows strict chain length specificity for C27–C31 substrates.

### NoCER1A Produces Shorter-Chain Alkanes in the *cer6* Mutant

**Fig. 5:**
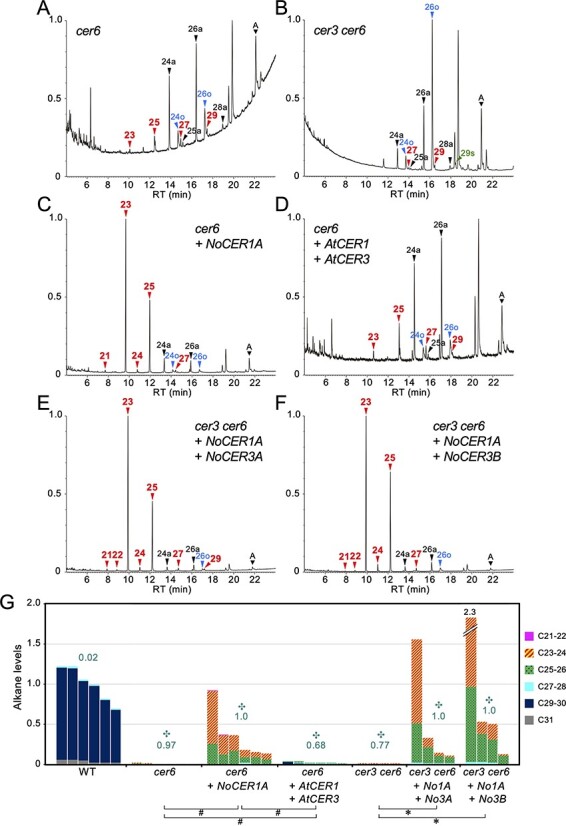
Production of short VLC alkanes in *cer6* and *cer3 cer6* mutants expressing *N. odorata* genes. **(A–F)** Total ion current chromatogram of GC-MS analysis. Typical results of *cer6* (A), *cer6* containing *ProAt1-NoCER1A* (B), *cer6* containing *ProAt1-AtCER1* and *ProAt3-AtCER3* (C), *cer3 cer6* (D), *cer3 cer6* containing *ProAt1-NoCER1A* and *ProAt1-NoCER3A* (E) and c*er3 cer6* containing *ProAt1-NoCER1A* and *ProAt1-NoCER3B* (F) are shown. **(G)** Quantification of alkane levels. Statistically significant differences of shorter alkane ratio from WT are indicated (✣, Steel’s test, *P *< 0.05). Relationships of total alkane levels with a statistically significant difference are shown (Steel’s test [✽] and Steel–Dwass test [#], *P *< 0.05). Other details are explained in **Fig. 3** legend.

Whereas *N. odorata* effectively produced C19–C23 alkanes in anthers, the transgenic Arabidopsis described earlier produced negligible amounts of these short alkanes. We assumed that the acyl-CoA substrates of C24 and shorter were less abundant in Arabidopsis stems, since these have a strong fatty acid elongase activity. Thus, we employed the *cer6* mutant, which lacks a fatty acid elongase required to produce C24–C28 acyl-CoAs (Haslam and Kunst 2013). We chose an allele in the Col-0 background, GABI_804G08, which has a T-DNA insertion in the first intron of the *CER6* gene, and named it *cer6-4* (**Supplementary Fig. S5**). Although this was not a null allele, we confirmed that the level of CER6 mRNA in the mutant was <1% of that in the WT (**Supplementary Fig. S10**). Consistent with previous reports (Fiebig et al. 2000, Goodwin et al. 2005), the *cer6* mutant not only produced only small amounts of C23–C29 alkanes but also produced relatively large amounts of C24 and C26 aldehydes and alcohols (**Fig. 5A and G** and **Supplementary Fig. S9**). When we introduced the *ProAt1-NoCER1A* gene into *cer6*, the transformants produced remarkable amounts of C23 and C25 alkanes as well as low but detectable levels of newly observed C21 and C24 alkanes. These data indicate that NoCER1A can produce short VLC alkanes in combination with endogenous Arabidopsis fatty acyl reductases (**Fig. 5C and G**). In a control experiment, the expression of *ProAt1-AtCER1* together with *ProAt3-AtCER3* in the *cer6* mutant, in which the endogenous *AtCER1* and *AtCER3* genes were functional, did not show altered wax composition, although alkane levels were slightly increased (**Fig. 5D and G**).

Next, we expressed both *ProAt1-NoCER1A* and *ProAt1-NoCER3A* (or *ProAt1-NoCER3B*) genes in *cer3 cer6* double mutants. These transformants predominantly produced C23 and C25 alkanes, and the wax composition was similar to that of the *ProAt1-NoCER1A* transformants in the *cer6* background (**Fig. 5E****–****G**). Similar characteristics were observed in T2 progeny of the *ProAt1-NoCER1A* and *ProAt1-NoCER3B* transformants, although their alkane levels were lower than those of the parental T1 plants (**Supplementary Fig. S9**). Based on these results, we hypothesized that NoCER1 worked together with NoCER3A, NoCER3B and AtCER3 to produce short VLC alkanes in Arabidopsis. However, wax quantification revealed that the levels of C24 and C26 aldehydes did not significantly differ between the *cer6* and *cer3 cer6* double mutants; moreover, more C26 alcohol was produced in the latter (**Supplementary Fig. S9**). This result indicated that C24 and C26 acyl-CoAs accumulated by defective CER6 elongase were efficiently converted to aldehydes and alcohols and that AtCER3 was not primarily responsible for this reaction. Accordingly, it cannot be ruled out that alternate endogenous reductase activity in Arabidopsis (i.e. other than AtCER3) may contribute to shorter alkane production in combination with NoCER1. Hence, we decided to use cultured tobacco cells to examine the contribution of NoCER3 to short VLC alkane production.

### Co-expression of *NoCER1A* and Any of the *CER3* Genes is Sufficient for Alkane Production in Cultured Tobacco Cells

**Fig. 6:**
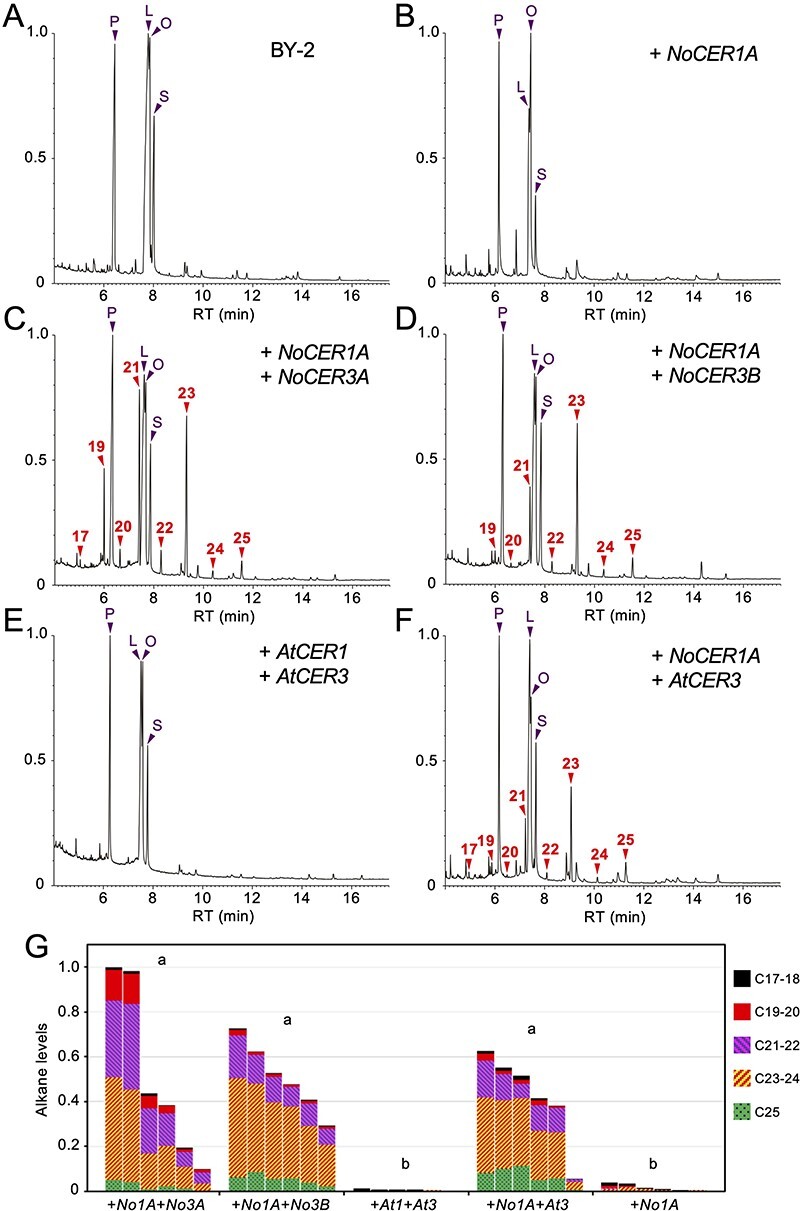
Alkane production in tobacco BY-2 cells expressing *NoCER1A* and various *CER3* genes. (**A–F**) Total ion current chromatogram of the GC-MS analysis. Typical results of a non-transformed BY-2 callus (A) and BY-2 calli containing *Pro35S-NoCER1A* alone (B), *Pro35S-NoCER1A* and *Pro35S-NoCER3A* (C), *Pro35S-NoCER1A* and *Pro35S-NoCER3B* (D), *Pro35S-AtCER1* and *Pro35S-AtCER3* (E) and *Pro35S-NoCER1A* and *Pro35S-AtCER3* (F) are shown. The vertical axis shows the relative intensity when the maximum peak is set as 1.0. Alkane peaks are labeled with red numbers indicating carbon chain length. P, palmitic acid; L, linoleic acid; O, oleic acid; S, stearic acid. (**G**) Total alkane levels in six independently obtained transformants expressing indicated genes. Ranges of carbon chain lengths are shown in different colors. Levels are shown in arbitrary units. Significant differences determined by Tukey’s test (*P* < 0.05) are indicated by letters above the bars.

Untransformed calli of BY-2 tobacco-cultured cells grown on agar plates did not produce detectable amounts of wax components but accumulated abundant C16 and C18 fatty acids (**Fig. 6A and G**). Introduction of *NoCER1A* driven by a cauliflower mosaic virus 35S promoter (*Pro35S*) did not by itself cause the production of any alkanes and aldehydes; the result confirmed that NoCER1A alone could not produce these compounds and that the cells lack endogenous reductase activities that work together with NoCER1A (**Fig. 6B and G**). Next, to test whether the coexpression of *NoCER1A* and *NoCER3A/B* is sufficient to synthesize alkanes, we introduced both *Pro35S-NoCER1A* and *Pro35S-NoCER3A* into the BY-2 cells. The transformants produced alkanes between C17 and C25 in length, and C19, C21 and C23 species were the most abundant (**Fig. 6C and G**). The introduction of the *Pro35S-NoCER1A* and *Pro35S-NoCER3B* pair provided a similar result, except that the abundance of the C17 and C19 alkanes decreased (**Fig. 6D and G**). The alkane levels in the transformants reached 43–53 μg/g fresh cell weight (**Supplementary Fig. S11**), which was calculated to be 1.0–1.3 mg/g dry cell weight. A control experiment introducing *Pro35S-AtCER1* and *Pro35S-AtCER3* did not produce any alkanes in BY-2 cells (**Fig. 6E and G**). Nevertheless, cells expressing *NoCER1A* and *AtCER3* produced similar sets of alkanes, indicating that NoCER3 can be replaced by AtCER3 (**Fig. 6F and G**). Taken together, these results indicate that the coexpression of *NoCER1A* and any one of *NoCER3A, NoCER3B* or *AtCER3* was sufficient to produce alkanes in BY-2 cells. NoCER1A and CER3s catalyze reactions for a broad range of chain lengths of acyl substrates, whereas AtCER1 has strict substrate specificity for C28–C32 molecules that are efficiently produced in certain cells such as the Arabidopsis epidermis.

### Alkane Chain Length is Important for the Formation of Wax Crystals Required for Self-Cleaning

**Fig. 7:**
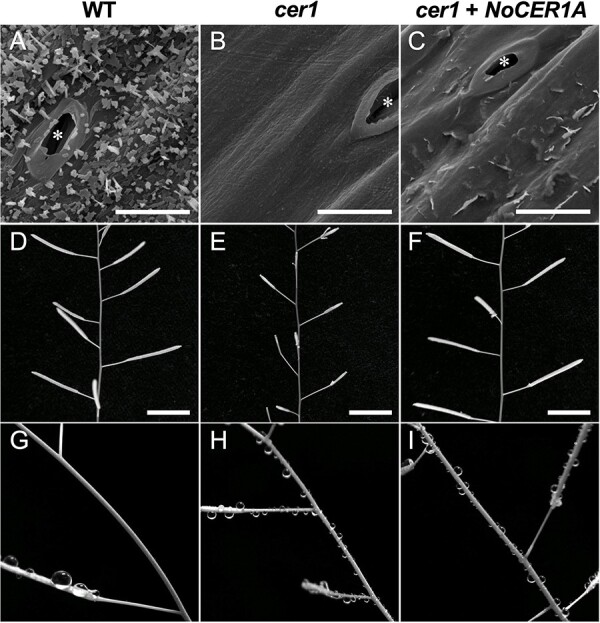
Epicuticular wax formation and pollen fertility in *NoCER1A*-expressing *cer1*. **(A–C)** Scanning electron micrographs of the stem surface in the WT (A), *cer1* (B) and *cer1* containing *ProAt1-NoCER1A* (C). Bar represents 10 μm. An asterisk denotes a stoma. **(D–F)** Fully developed self-pollinated fruits reflecting normal pollen fertility in the WT (D) and *cer1* containing *ProAt1-NoCER1A* (F) in comparison to unfertilized fruits in male-sterile *cer1* (E). The bar represents 10 mm. **(G–I)** Stems after spraying with water. The WT stem repelled water (G), whereas water droplets adhered to the stems of *cer1* (H), and *cer1* containing *ProAt1-NoCER1A* (I).

Scanning electron microscopy revealed that many wax crystals were deposited on the stem surface of WT Arabidopsis plants (**Fig. 7A**), while the waxless *cer1* mutant showed no such crystal formation as well as male sterility caused by defective pollen coat formation (**Fig. 7B and E**). Transgenic *cer1* plants containing *ProAt1-NoCER1A* showed a restored ability to produce C23–C29 alkanes in stems and to set self-pollinated seed, but irregular scales instead of wax crystals were formed on the stems (**Figs. 4B**, **7C**, and **F**). The amount of nonalkane wax components did not significantly differ between the *ProAt1-NoCER1A* and WT plants (**Supplementary Fig. S12**), indicating that mixed shorter alkanes (and possibly the reduced levels of alkane-derived secondary alcohols and ketone) inhibited the formation of a crystalline structure. Similar defects in the wax crystal formation were observed in *cer1 cer3* double mutants expressing the *ProAt1-NoCER1A* and *ProAt3i-NoCER3A* (or *ProAt3i-NoCER3B*) (**Supplementary Fig. S13A-D**), of which wax compositions were indicated in **Supplementary Fig. S9**. In contrast, introduction of the *ProAt1-AtCER1* and *ProAt3i-AtCER3* genes into *cer1 cer3* double mutants restored the ability to form wax crystals, even though the plants that produced less amounts of alkanes (**Supplementary Fig. S13E and F**). These results confirmed that the alkane composition rather than the alkane amount affects the formation of wax crystals.

Wax crystals are thought to be involved in a self-cleaning process called the lotus effect, by which water is repelled by the crystalline microstructure and forms droplets; these droplets wash away pollutants and pathogen spores deposited on the plant surface. Here, water sprayed onto WT plants caused an obvious lotus effect, and no water droplets remained on the stem surface (**Fig. 7G**). However, after spraying the surface of *NoCER1A*-expressing *cer1* plants and the untransformed *cer1* mutant, we observed that many water droplets adhered to the stem surface (**Fig. 7H and I**), confirming the hypothesis that the production of wax crystals is important for water shedding. Taken together, these results suggest that Arabidopsis acquired the activity to produce C29 alkane exclusively, likely to make self-cleaning more effective.

## Discussion

### Substrate Carbon Chain-Length Specificities of NoCER1A and NoCER3A/B

We found that water lilies produce alkanes with shorter carbon chain lengths than those produced by Arabidopsis, suggesting that some property of the alkane synthesis enzymes in water lilies directs them to produce shorter alkanes. We isolated full-length CDS clones for an *AtCER1* homolog (*NoCER1A*) and two *AtCER3* homologs (*NoCER3A* and *NoCER3B*) from this species. Complementation experiments in Arabidopsis *cer1* and *cer3* mutants revealed that *NoCER1A* and *NoCER3A/B* are functional counterparts of *AtCER1* and *AtCER3*, respectively. A recently published genome sequence of *N. colorata* revealed that it has two genes that closely resemble *NoCER1A* and two *CER3* genes that corresponded to *NoCER3A* and *NoCER3B*, respectively (Zhang et al. 2020). Hence it is suggested that the shorter alkanes in water lilies are produced by these enzymes. *CER1* and *CER3* are thought to be two separate genes formed by the functional differentiation of a single ancestral gene (Wang et al. 2019, Chaudhary et al. 2021). Phylogenetic analysis of the predicted amino-acid sequences indicated that NoCER1A, together with two *A. trichopoda* proteins, first branched off from other CER1 homologs in angiosperms, which is consistent with the evolutionary position of water lilies. Similar relationships were observed for CER3 proteins.

Introduction of *NoCER1A* restored alkane production in the Arabidopsis *cer6* mutant, in which C28 and longer acyl-CoAs could not be synthesized. Although AtCER1 was active in the mutant, it could not use C26 or shorter substrates. Similar results were obtained from the experiments using tobacco BY-2 cells, where NoCER1A but not AtCER1 produced C25 and shorter alkanes in combination with either NoCER3A/B or AtCER3. Moreover, NoCER1A effectively produced C29 alkane, when it was introduced into the *cer1* mutant. These results show an unequivocal difference between AtCER1 and NoCER1A in that the former preferentially produces C29 alkanes, whereas the latter synthesizes C19–C29 alkanes nonselectively. This indicates that AtCER1 but not NoCER1A shows strict carbon chain-length specificity for fatty-aldehyde substrates.

A similar chain-length specificity was observed in the activity of the midchain alkane hydroxylase, where the C29 alkane was effectively converted to C29-15-alcohol and C29-15-ketone, but a negligible amount of C27 and shorter ketones and secondary alcohols were measured even when NoCER1A produced a considerable amount of shorter alkanes (**Figs. 3** and **4**) (Greer et al. 2007).

### Predicted 3D Structures of NoCER1A and NoCER3A/B

Despite the low-sequence similarity between NoCER1A and AtCER1, the predicted 3D structures of NoCER1A (A0A7G1GB01) and AtCER1 (F4HVY0) recorded by the AlphaFold Protein Structure Database closely resembled each other (**Supplementary Fig. S14A and B**). Each consisted of an N-terminal domain (NTD) comprising His-rich motifs accumulated in a small region coupled with six parallelly aligned helices and a C-terminal domain (CTD) that includes the WAX2 domain. According to homology modeling on the SWISS-MODEL server, the NTD resembles the catalytic domain of yeast sphingolipid α-hydroxylase, Scs7p (4ZR1), which hydroxylates C-2 of a VLC fatty acid moiety. This domain comprises a His-rich active site and four transmembrane helices to form a lipid-binding channel, which anchors the enzyme to the ER (**Supplementary Fig. S14C**) (Zhu et al. 2015). The arrangement of CER1 proteins is such that four helices of the six lay on the clustered His residues, and this is coincident with the characteristics of Scs7p (**Supplementary Fig. S14D–G**). We therefore speculate that the NTD is the catalytic domain of CER1 proteins and the four helices on the His-rich motifs form a lipid-binding channel embedded in the membrane. It has been proposed that a specific amino-acid residue in the lipid-binding channel of Scs7p may help determine the carbon chain-length specificity of the substrate (Zhu et al. 2015). Although the amino-acid sequences of the four helices forming the putative lipid-binding channel are not similar in NoCER1A and AtCER1 (**Supplementary Fig. S1**), the 3D structures of this region of the two proteins are nearly identical. Hence, it is difficult to attribute differences in the carbon chain-length specificity of these proteins to structural differences between their putative catalytic domains.

Previous studies have elucidated that the cyanobacteria employ an acyl–acyl carrier protein reductase (AAR) to convert fatty acyl groups into fatty aldehydes for synthesizing alkanes (Schirmer et al. 2010). Although sequence identity scores between CER3s and AARs were very low (Bernard et al. 2012), a CTD structural model of NoCER3A (A0A7G1G966 in the AlphaFold Database) clearly resembled the cleft region of *Synechococcus elongatus* PCC7942 ARR (SeARR; PDB code 6JZQ) (**Supplementary Fig. S15A–C**), which has a 3D structure that was determined via X-ray crystallography (Gao et al. 2020). The region was shown to constitute a substrate entrance to the catalytic center and a binding site for NADPH. The catalytic residue, C294, is located at the bottom of the cleft. The overlaid structure revealed that a Cys residue (C577) of NoCER3A was located at the same position as C294 in SeAAR, suggesting that the C577 may be the catalytic residue of NoCER3A (**Supplementary Fig. S15A-C**). This hypothesis is consistent with the conservation of the Cys residue in CER3 proteins but not in CER1 proteins (**Supplementary Figs. S1, S2 and S15D**). The NoCER3A model was almost completely overlaid on the structure of AtCER3 (Q8H1Z0) and NoCER3B (A0A7G1GAG9), which further suggests that the 3D structure is common to all CER3 proteins (**Supplementary Fig. S15E–H**). Furthermore, the structures of CER1s and CER3s also highly resemble each other (**Supplementary Fig. S16**).

Despite overall similarity in the structure of CER1 and CER3, the active site is located in different positions in CER1 (at the NTD) and CER3 (at the CTD). This is consistent with the relatively high conservation of the NTD in CER1s and the low conservation of this region in CER3s (and vice versa for the CTD). Recent studies have shown that the ancestral enzyme of CER1 and CER3 was the result of a fusion of two enzymes that differ in both structure and activity (Chaudhary et al. 2021). During subsequent evolution, each may have changed such that only one of the domains became or remained active.

**Fig. 8:**
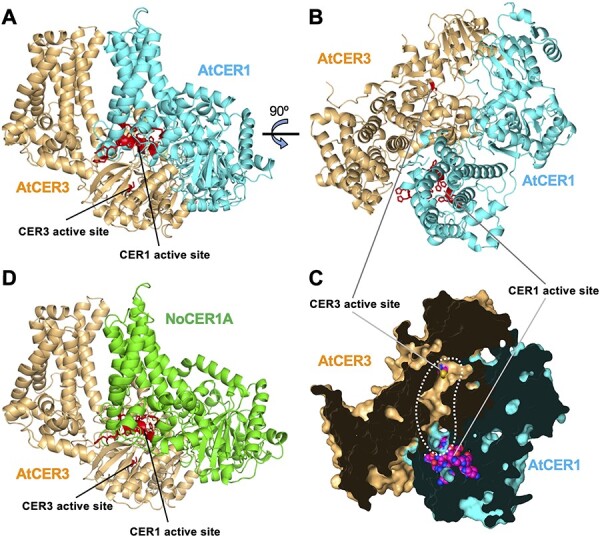
Predicted 3D structures of CER1 and CER3 complexes. **(A–C)** A structure of AtCER1 and AtCER3 complex predicted by AlphaFold2_advanced. (A) Side view. (B and C) Top view. In (A) and (B), His residues in the tripartite His clusters in AtCER1 and the Cys residue in AtCER3 active site are shown in red. (C) Cutaway view of the putative substrate tunnel (dotted line) connecting the two active sites, of which His and Cys atoms are indicated by spheres. **(D)** A predicted structure of NoCER1A and AtCER3 complex.

We showed that *N. odorata* CER1 and CER3 rescued Arabidopsis *cer1* and *cer3* mutants, respectively, which suggests that *N. odorata* proteins performed their activities in combination with Arabidopsis AtCER1 and AtCER3. Since it has been shown that AtCER1 physically interacts with AtCER3 (Bernard et al. 2012), *N. odorata* and Arabidopsis proteins are thought to be able to form enzymatic complexes. To predict the complex structures of CER1 and CER3, we used AlphaFold2_advanced. The model thus generated showed that AtCER1 and AtCER3 were aligned in the same direction and the NTD of AtCER1 and the CTD of AtCER3 were bound tightly (**Fig. 8A, B**). Moreover, an internal view of the complex revealed that the putative active sites of CER3 and CER1 were connected by a cavity that extends to the predicted lipid-binding channel of CER1 (**Fig. 8C**). We therefore speculate that this predicted structure explains the synergistic function of AtCER1 and AtCER3 in alkane production, although no experimental evidence confirming this is currently available. This prediction also suggests that NoCER1A and AtCER3 could form a complex with almost the same structure (**Fig. 8D**), which is coincident with the cooperative function of NoCER1A and AtCER3 in alkane production. The large substrate-binding cavity may therefore account for the lack of carbon chain-length specificity in CER3 proteins.

### Chain-Length Specificity Contributes to Wax Crystal Formation

In contrast to the C29-preferential alkane production in WT stems, *NoCER1A*-expressing *cer1* plants produced shorter alkanes (i.e. as small as C23), even though the plants possessed functional fatty acid elongases. This indicated that CER1 and CER3 compete with elongase for acyl-CoA substrates in the ER. Incorporation of shorter-chain alkanes into the stem epicuticular wax hindered the crystal formation required for self-cleaning. Similar defects in wax crystal formation have been observed when the amount of C31 alkane exceeded the level of C29 alkane caused by ectopic expression of CER2-LIKE1/CER26 (Pascal et al. 2013, Haslam et al. 2015). Moreover, increased C23 and C25 alkanes in plants overexpressing CER1-LIKE1 also prevented crystallization (Pascal et al. 2019). Therefore, we suggest that the chain-length specificity of C29 alkane production is important to prevent competition with elongase for shorter-chain substrates and may have been acquired during the evolution of Arabidopsis. In contrast, *N. odorata* does not require such enzymes, since it does not produce wax crystals on its organ surfaces. Instead, it has been reported that the alkanes (and alkenes) ranging from C14 to C21 are major components of flower volatiles in water lilies (Yuan et al. 2014, Tsai et al. 2019). These suggest that the alkanes with different carbon chain lengths have a variety of uses in different plant species. It should be noted that the pollen fertility of *cer1* was completely restored by the expression of the *NoCER1A* gene, presumably because no crystal formation is required for the function of the pollen coat. Furthermore, the development and growth of Arabidopsis plants with altered alkane composition generated in this study were normal at least in our laboratory condition.

### Function and Regulation of CER3 in Aldehyde Production

In plants, there exist two types of acyl-CoA reductases. One type comprises aldehyde-generating enzymes such as CER3. The other type comprises alcohol-generating enzymes that catalyze two-step reactions forming primary alcohols via an aldehyde intermediate, which remains bound to the enzymes (Metz et al. 2000, Doan et al. 2009). In Arabidopsis, the latter includes fatty acyl-coenzyme A reductase1 (FAR1) to FAR8. It has been shown that FAR3/CER4 produces VLC alcohols in epicuticular waxes (Rowland et al. 2006). FAR1, FAR4 and FAR5 have been found to be involved in suberin formation and FAR2/MS2 is important for sporopollenin formation (Aarts et al. 1997, Dobritsa et al. 2009, Domergue et al. 2010). Even though the *cer3* allele used in our experiments was a null allele, the *cer3* mutants produced considerable amounts of alkanes, suggesting that an alternate reductase—i.e. other than AtCER3—provided aldehyde intermediates for alkane production. Since AtCER3 is currently thought to be a unique aldehyde-generating reductase in Arabidopsis, these aldehyde intermediates may be provided by an unidentified enzyme, or possibly by FAR.

While aldehydes accumulated in *cer1*, these levels were much lower than the amount of alkanes present in the WT, even though AtCER3 was active in the mutant (**Supplementary Fig. S9**). Similarly, the aldehyde levels remained low in *cer6*, in which both AtCER1 and AtCER3 were active, but the former could not work since the substrates with appropriate carbon chain lengths were never supplied. Taken together, these results suggest that AtCER3 activity was suppressed when AtCER1 was not functional. This may reflect the cooperative function of AtCER1 and AtCER3 in their complex. Alternatively, plants may have a system to suppress the accumulation of toxic aldehydes.

### Introduction of NoCER1A and one of the CER3 is Sufficient for Alkane Production in Plant Cells

We found that the introduction of only two genes, *NoCER1A* and any one of the *CER3*s, is sufficient to synthesize C17–C25 alkanes in tobacco BY-2 cells, which do not otherwise produce a detectable amount of alkanes without genetic modification. Our findings therefore show that BY-2 cells can produce VLC acyl-CoA molecules of approximately this range in the ER and that these molecules are available to NoCER1A for alkane production, whereas they are too short for AtCER1. In many plant leaves, C20–C26 VLC fatty acids are synthesized and used to produce sphingolipids and some classes of phospholipids (Bach and Faure 2010). Therefore, it is expected that the expression of NoCER1A and an appropriate CER3 will facilitate the production of VLC alkanes in many plant cells. In addition, our BY-2 expression analysis also revealed slight differences in chain-length specificity among CER3 proteins. NoCER3A uses short acyl-CoA substrates (especially C20 and C22) more effectively than others.

The alkane levels produced by transgenic BY-2 cells reached 43–53 μg/g fresh cell weight (or 1.0–1.3 mg/g dry cell weight). Although this yield is 1/500 of the recent record of alkane production in oleaginous yeast *Yarrowia lipolytica* (Bruder et al. 2019, Hu et al. 2019), we provide evidence of alkane production in a heterologous system using water lily alkane-forming enzymes. These BY-2 cells accumulate alkanes presumably in the ER since they lack any specific lipid-storage organelles, but if they develop those organelles, such as tapetosome in anther tapetal cells, they may accumulate more alkanes in the cells. Bernard et al. (2012) demonstrated that AtCER1 and AtCER3 require VLC fatty acid elongase for alkane production in yeast and that the resulting product was mainly C29 alkane supplemented with a small amount of C27–C31 alkanes. This composition clearly reflected the chain-length specificity of AtCER1. Yeast can produce VLC fatty acids up to C26 in length (Welch and Burlingame 1973). Hence, it is expected that the expression of NoCER1 together with an appropriate CER3 can produce VLC alkanes in yeast without requiring the use of VLC fatty acid elongases.

## Materials and Methods

### Plant Materials and Growth Conditions

Fragrant water lily (*N. odorata*) grown in a pond of Nagoya University Museum Botanical Garden was used as the source of pollen-coat lipid and anther RNA. Arabidopsis (*Arabidopsis thaliana*) *cer1* (*cer1-1*; SALK_008544) (Bourdenx et al. 2011), *cer3* (*cer3-9*; GABI_177D09) (Xu et al. 2020) and *cer6* (*cer6-4*; GABI_804G08) (established in this paper) mutants were obtained from the Arabidopsis Biological Resource Center and Nottingham Arabidopsis Stock Centre (**Supplementary Fig. S6**). All the wild-type and mutant Arabidopsis plants used in this study were of Col-0 accession and were grown on vermiculite at 22°C under continuous illumination by fluorescent light. Tobacco (*Nicotiana tabacum*) suspension-cultured cell line BY-2 was maintained in a liquid BY-2 medium (Murashige and Skoog plant salt mixture, 3% sucrose, 200 mg/L KH_2_PO_4_, 100 mg/L *myo*-inositol, 1 mg/L thiamine HCl, 0.2 mg/L 2,4-dichlorophenoxyacetic acid, pH 5.8) with constant rotation (110 rpm) at 25°C under darkness.

### Plant Transformation

Transformation of Arabidopsis was carried out as described previously (Ishiguro et al. 2010). To obtain the transformants in *cer3, cer1 cer3* and *cer3 cer6* backgrounds, heterozygotes for *cer3* mutation were used for *Agrobacterium* infection because *cer3* homozygotes were strongly sterile. The T1 transformants homozygous for *cer3* mutation were selected by PCR genotyping. For transformation of BY-2 cells, 4 mL of 3-day-old BY-2 suspension culture was poured into a 9 cm diameter dish and mixed with 100 μL of fully grown *Agrobacterium* culture harboring the binary plasmid described further. After static co-cultivation for 2 days at 25°C, cells were washed with liquid BY-2 medium and scattered on a plate of BY-2 medium solidified with 0.4% gellan gum and 0.1% MgSO_4_ · 7 H_2_O and supplemented with 50 mg/L hygromycin B and 500 mg/L cefotaxime. After cultivation for 3–4 weeks at 25°C, transformed calli were harvested and used for lipid analysis.

### RNA Extraction

*Nymphaea odorata* anthers (200 mg) containing developing tapetal cells were harvested, frozen in liquid nitrogen and powdered together with 1 mL TRIzol Reagent (Thermo-Fisher Scientific) using a frozen mortar and pestle. The powder was transferred to a preheated (70°C) mortar and further ground with preheated 1 mL TRIzol Reagent. After adding 400 μL chloroform, the crude RNA was collected from the aqueous phase by isopropanol precipitation. The RNA was purified using an RNeasy plant mini kit (Qiagen, Venlo, Netherlands). Arabidopsis RNA in the flower bud clusters was both extracted and purified using an RNeasy plant mini kit.

### Reverse Transcription and Degenerate PCR

*Nymphaea odorata* anther RNA (0.5 μg) was reverse transcribed with ReverTra Ace qPCR Master Mix with gDNA Remover (Toyobo, Osaka, Japan). The resulting first-strand cDNA was used as a template for PCR amplification with the degenerate *CER1* and *CER3* primers (**Supplementary Table S3**) using PrimeSTAR HS DNA polymerase (Takara Bio, Kusatsu, Japan). PCR products were cloned in the *Sma*I site of pUC119, and eight independently obtained clones were sequenced.

### NGS Analysis

A cDNA library was constructed from 0.5 μg anther RNA using a TruSeq RNA Sample Preparation Kit (Illumina, San Diego, CA, USA) and was used for paired-end sequencing for 60 cycles by Illumina Genome Analyzer IIx. Sequence reads were de novo assembled into contigs by Trinity software (Grabherr et al. 2011).

### Preparation of Full-Length CDS Clones

All the primers used for cloning and plasmid construction are listed in **Supplementary Table S3**. Full-length CDS for *NoCER1A, NoCER3A* and *NoCER3B* were amplified by PCR from water lily anther cDNA as described earlier. Primers were designed according to the NGS data. The CDS fragments were cloned in the *Sma*I site of pUC119. Resulting plasmids were designated as pUC-NyCER1A, pUC-NyCER3A and pUC-NyCER3B. The full-length CDS fragments of *AtCER1* and *AtCER3* were prepared by RT-PCR from Arabidopsis young flower bud RNA and cloned into pDONR201 by Gateway BP reaction (Thermo-Fisher Scientific, Waltham, MA, USA). They were named as pDONR-AtCER1 and pDONR-AtCER3.

### Phylogenetic Analysis

Maximum-likelihood phylogenetic trees were constructed using MEGA11 software with 1000 bootstrap replicates (Tamura et al. 2021). The sequences used to make the trees are listed in **Supplementary Table S2**.

### Construction of Plasmids for *CER1* and *CER3* Gene Expression

All plant transformation plasmids for *CER* gene expression were prepared by way of the Gateway recycling cloning system (Kimura et al. 2013). Details are described in the Supplementary Methods.

### Lipid Extraction

Anthers harvested from fully opened flowers were immersed in water in a microcentrifuge tube and vortexed vigorously. Suspended pollen grains were collected by centrifugation, and their surface lipid (pollen coat) was extracted with a small volume of chloroform (300 μL). After evaporation of solvent by N_2_ gas flow, the deposited pollen coat was dissolved in hexane and served for GC-MS analysis.

To extract stem cuticular wax, a cut piece of the basal part (3 cm in length) of the lateral shoot branched from the main shoot was harvested into a microcentrifuge tube. A small volume of chloroform (200 μL) was added into the tube, and lipid was extracted with gentle mixing for 30 s. After evaporation of solvent by N_2_ gas flow, the precipitated lipid was dissolved in hexane and served for GC-MS analysis. To quantify the absolute alkane levels, 300 ng of C15 alkane was added to the samples at chloroform extraction as an internal standard.

A part (10–30 metreg) of BY-2 calli grown on the solidified medium was harvested into a microcentrifuge tube. A small volume (300 μL) of chloroform/methanol (1/1) was added to the cells, and vigorous vortexing was performed for 1 min to extract lipids. After centrifugation to remove cells, the extract was dried with N_2_ gas flow and dissolved in hexane for GC-MS analysis. To quantify the absolute alkane levels, 300 ng of C15 alkane was added to the samples at chloroform/methanol extraction as an internal standard.

### GC-MS Analysis

Lipids were analyzed by a mass spectrometer, JMS-K9 (JEOL), equipped with a DB-1 column (30 m × 0.25 mm ID × 0.25 μm film) (Agilent Technologies, Santa Clara, CA, USA) under the following parameters: He gas flow, 1.5 mL/min; oven temperature, 50–200°C (50°C/min) and then 200–300°C (5°C/min). Separated samples were ionized by electron ionization at 70 eV, and a *m/z* range of 50–500 was detected. Representative mass spectrum data of each type of wax components were indicated (**Supplementary Fig. S17**). We used β-amyrin and palmitic acid as endogenous internal standards for the quantification of alkanes in Arabidopsis stem waxes and in BY-2 cells, respectively. For absolute quantification of wax components, amounts were normalized by the levels of exogenously added C15 alkane and were corrected by relative response factors determined in our condition (0.19 for primary alcohols and 1.0 for aldehydes). For statistical analysis, we used the non-parametric U-test, Steel’s test and Steel–Dwass test for alkane quantification in T1 plants because some data did not follow a normal distribution. For the data of T2 plants and BY-2 cells, we used the parametric Tukey’s test or Tukey–Kramer test.

### Scanning Electron Microscopy

A cut piece of the basal part of lateral stems that branched off from the main shoot was attached on an aluminum stab with double-sided adhesive tape and coated with Au in an ion sputter (MSP-1S, Vacuum Device). The specimen was observed by a scanning electron microscope (S-2600 N, Hitachi High-Tech, Tokyo, Japan) at an accelerating voltage of 10 kV in high vacuum mode.

### Prediction of Protein Structures

The modeling tool in SWISS-MODEL server (https://swissmodel.expasy.org/) was used to find a template of CER1 NTD. PDB files of proteins were obtained from PDB database (https://www.rcsb.org/) and AlphaFold Protein Structure Database (https://alphafold.ebi.ac.uk/) and visualized by PyMOL software (https://pymol.org/). Structures of CER1 and CER3 complex were modeled by AlphaFold2_advanced software (https://colab.research.google.com/github/sokrypton/ColabFold/blob/main/beta/AlphaFold2_advanced.ipynb).

## Supplementary Material

pcad168_Supp

## Data Availability

The following data are available in the DDBJ/EBI/NCBI databases. Nucleotide sequences of CDS clones: NoCER1A (LC422236), NoCER3A (LC422237), NoCER3B (LC422238). FASTQ file of the NGS reads of *N. odorata* anther RNA: DRA007258.
