# Supplementary material for: Broad Chain-Length Specificity of the Alkane-Forming Enzymes NoCER1A and NoCER3A/B in Nymphaea odorata
Source: Plant Cell Physiol. 2024 Feb 9;65(3):428–46. doi: 10.1093/pcp/pcad168 (PMC11020225; doi:10.1093/pcp/pcad168)
Supplement: pcad168_Supp [file pcad168_supp.zip › pcad168_Supp/suppl_data/NyCER1CER3_Suppl Fig 240122proof2.pdf]

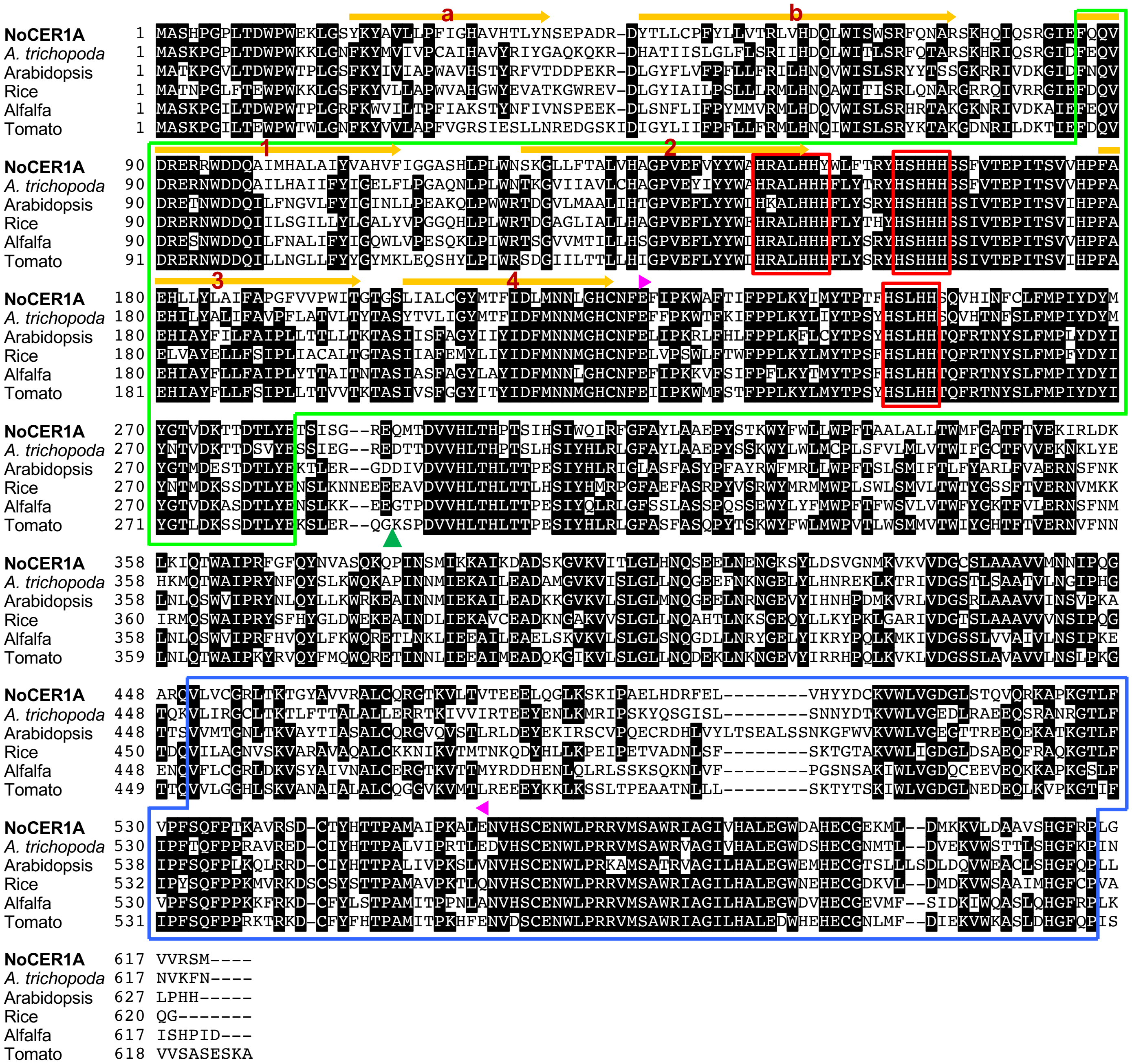

Supplementary Fig. S1

Predicted sequences of the proteins encoded by *NoCER1A* and its putative orthologs in angiosperms.

Comparison among predicted full-length amino-acid sequences of NoCER1A and representative closely homologous proteins in *A. trichopoda* (AmtrCER1A), Arabidopsis (AtCER1), rice (OsGL1-5), alfalfa (Medtr8g009560), and tomato (Solyc03g065250). Identical amino-acid residues among at least four sequences are outlined. The N-terminal putative lipid-binding and catalytic domain including tripartite His clusters (red boxes) and the C-terminal WAX2 domain are outlined by green and blue lines, respectively. Orange arrows represent the positions of putative transmembrane helices and the green triangle indicates the boundary between NTD and CTD (see Supplemental Figure S13). The region between the two magenta arrowheads corresponds to the sequences of DPCR.

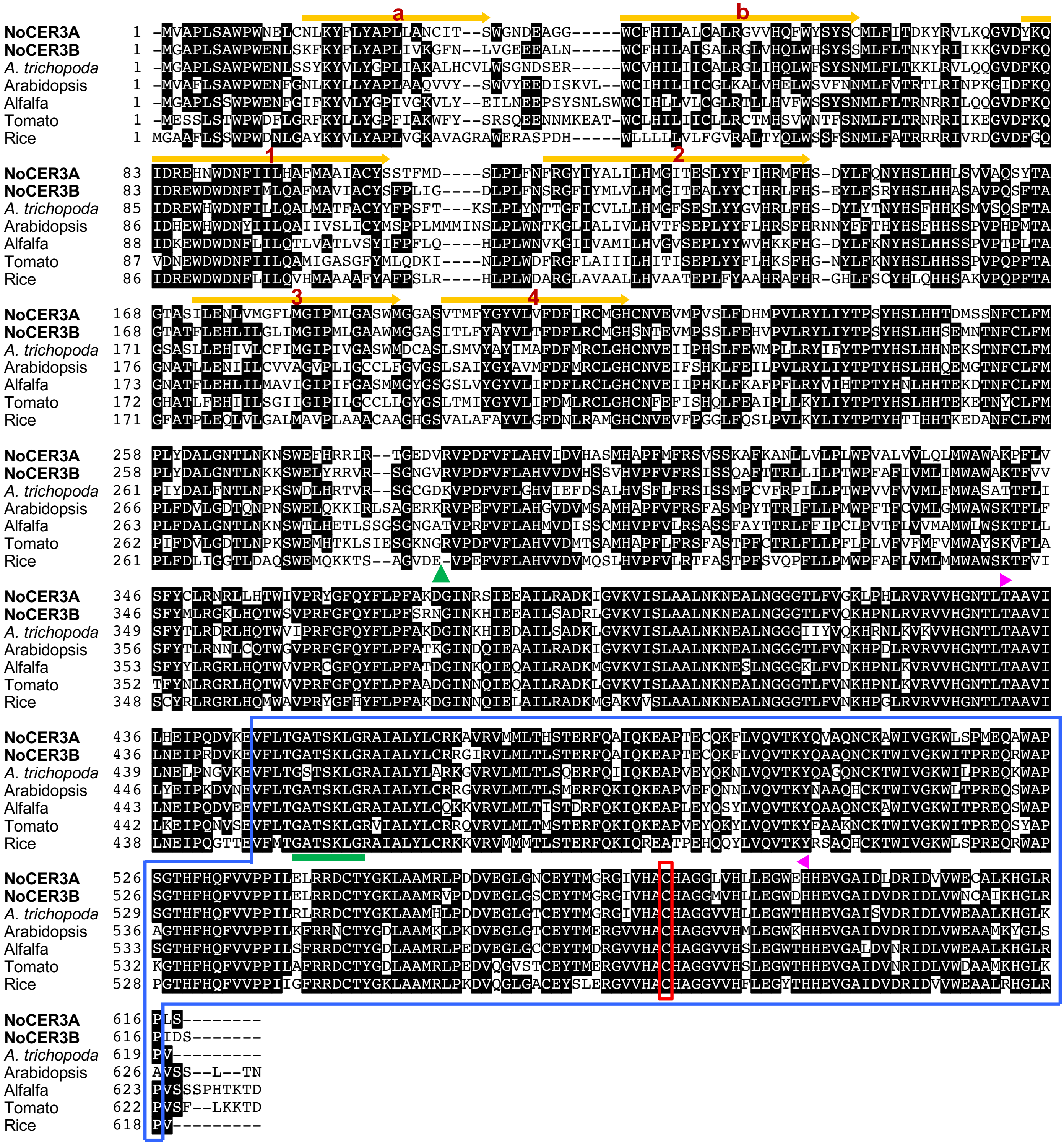

Supplementary Fig. S2

Predicted sequences of the proteins encoded by *NoCER3A/B* and their putative orthologs in angiosperms.

Comparison among predicted full-length amino-acid sequences of NoCER3A, NoCER3B, and representative closely homologous proteins in *A. trichopoda* (AmtrCER3A), Arabidopsis (AtCER3), alfalfa (Medtr4g129630), tomato (Solyc07g006300), and rice (OsGL-1). Identical amino-acid residues among at least five sequences are outlined. The WAX2 domain is outlined by blue lines. The putative catalytic cysteine residues (red box) and the region corresponding to the NADPH binding site in SeAAR (green underline) are indicated. The orange arrows and the green triangle are the same as Supplemental Figure S1. The region between the two magenta arrowheads corresponds to the sequences of DPCR.

CER1

CER1	No	Amtr		Arabidopsis			Rice			Alfalfa							Tomato				
	NoCER1A	AmtrCER1A	AmtrCER1B	AtCER1	AtCER1-LIKE1	AtCER1-LIKE2	OsGL1-4	OsGL1-5	OsGL1-6	Medtr4g054150	Medtr4g054290	Medtr7g090100	Medtr7g090120	Medtr7g090140	Medtr8g009560	Medtr8g009590	Solyc01g088400	Solyc01g088430	Solyc03g065250	Solyc08g044260	Solyc12g100270
NoCER1A	100																				
AmtrCER1A	62.3	100																			
AmtrCER1B	57.9	61.2	100																		
AtCER1	50.0	56.0	55.6	100																	
AtCER1-LIKE1	50.2	49.8	52.3	57.2	100																
AtCER1-LIKE2	50.4	52.5	52.5	56.3	66.8	100															
OsGL1-4	50.5	54.9	55.8	56.6	52.6	52.9	100														
OsGL1-5	52.8	55.8	56.5	58.4	55.0	54.9	62.9	100													
OsGL1-6	49.8	51.0	52.1	52.8	50.0	50.6	53.0	53.7	100												
Medtr4g054150	47.8	51.2	52.2	53.5	53.8	52.9	52.0	53.0	50.0	100											
Medtr4g054290	50.0	51.9	55.4	56.1	54.8	54.7	54.7	55.0	52.2	87.1	100										
Medtr7g090100	51.3	53.5	57.8	58.2	56.7	56.2	55.5	56.6	53.1	78.4	82.0	100									
Medtr7g090120	51.5	53.3	56.4	57.4	56.5	55.7	54.9	56.8	52.8	77.1	81.2	89.6	100								
Medtr7g090140	50.1	52.9	56.5	56.6	57.5	55.8	55.2	54.8	52.2	77.9	82.0	88.3	87.0	100							
Medtr8g009560	52.1	54.3	58.1	61.3	57.2	57.0	55.7	56.9	54.7	62.7	64.1	65.7	64.9	64.6	100						
Medtr8g009590	54.5	56.0	57.4	62.6	58.1	58.9	58.2	60.0	54.6	57.9	60.5	61.8	61.5	60.8	64.2	100					
Solyc01g088400	53.6	56.2	58.4	62.5	57.1	58.2	59.9	61.9	53.7	58.8	61.3	63.8	63.6	62.4	66.5	69.3	100				
Solyc01g088430	53.5	56.1	56.1	59.4	55.0	58.4	58.9	59.5	51.9	58.1	60.4	61.8	61.8	61.2	63.0	66.2	77.9	100			
Solyc03g065250	53.7	56.3	58.0	64.8	58.8	59.0	59.9	61.2	53.7	60.0	62.2	63.4	63.8	62.8	65.9	68.8	78.9	75.3	100		
Solyc08g044260	53.6	50.6	62.4	73.1	66.8	55.1	65.6	63.7	54.2	54.0	56.4	66.5	67.1	65.6	71.2	73.1	80.1	73.5	98.1	100	
Solyc12g100270	53.4	54.0	54.8	58.2	55.4	57.4	59.5	58.6	50.4	56.7	58.2	60.0	60.1	58.8	61.6	62.6	76.0	74.1	73.6	66.4	100

CER3

CER3	No		Amtr		Arabidopsis	Alfalfa	Rice			Tomato	
	NoCER3A	NoCER3B	AmtrCER3A	AmtrCER3B	AtCER3	Medtr4g129630	OsGL1-1	OsGL1-2	OsGL1-3	Solyc03g117800	Solyc07g006300
NoCER3A	100										
NoCER3B	76.6	100									
AmtrCER3A	68.2	72.2	100								
AmtrCER3B	59.8	61.7	61.2	100							
AtCER3	62.8	65.6	65.0	56.8	100						
Medtr4g129630	64.9	67.8	67.6	58.1	67.6	100					
OsGL1-1	61.6	63.0	59.4	56.4	63.5	64.8	100				
OsGL1-2	59.9	61.4	58.6	55.5	60.8	64.3	68.7	100			
OsGL1-3	61.2	64.7	59.8	58.7	62.0	65.2	67.7	79.4	100		
Solyc03g117800	64.0	66.5	63.8	56.6	65.4	66.0	62.4	62.4	62.6	100	
Solyc07g006300	62.5	65.8	65.0	58.5	69.9	70.4	62.5	61.2	61.6	66.2	100

Identity (%)
- 90 ≤
- 80-90
- 70-80
- 60-70
- 55-60
- 50-55
- < 50

Supplementary Fig. S3
Sequence identities among CER1 and CER3 homologs in *N. odorata* (No), *A. trichopoda* (Amtr), Arabidopsis, rice, alfalfa, and tomato.
Identities (%) among the predicted full-length protein sequences were calculated and color coded according to the scores.

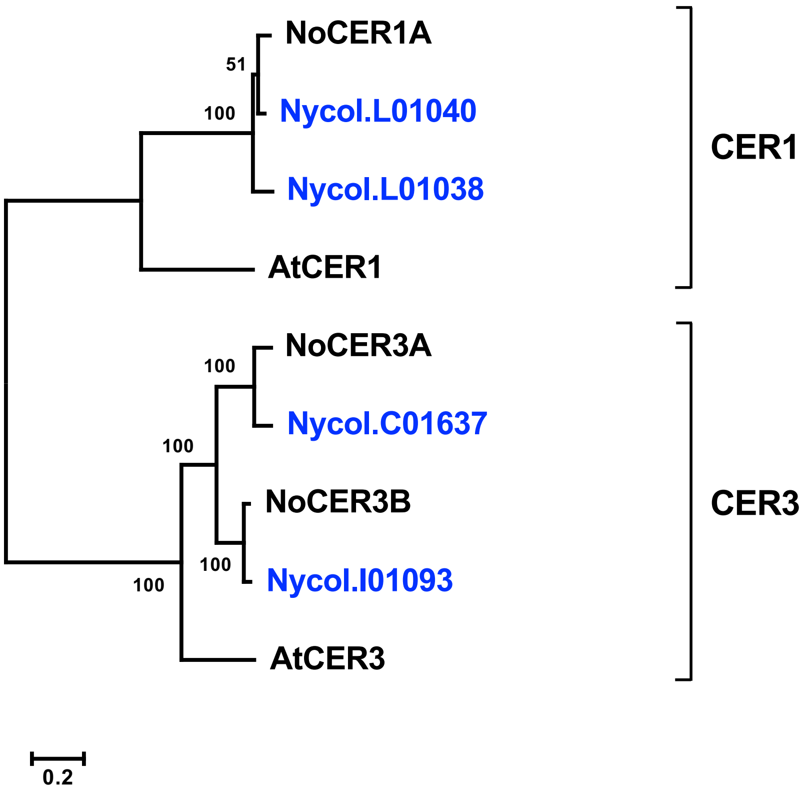

Supplementary Fig. S4
Structural relationship among CER1 and CER3 orthologs in *N. odorata* and *N. colorata*.
Maximum likelihood phylogenetic tree for comparing the amino-acid sequences of *N. odorata* and *N. colorata* CER1 and CER3. *N. colorata* proteins are indicated by blue. Bootstrap support values from 1000 replicates are indicated. The bar represents 0.2 substitutions per site.

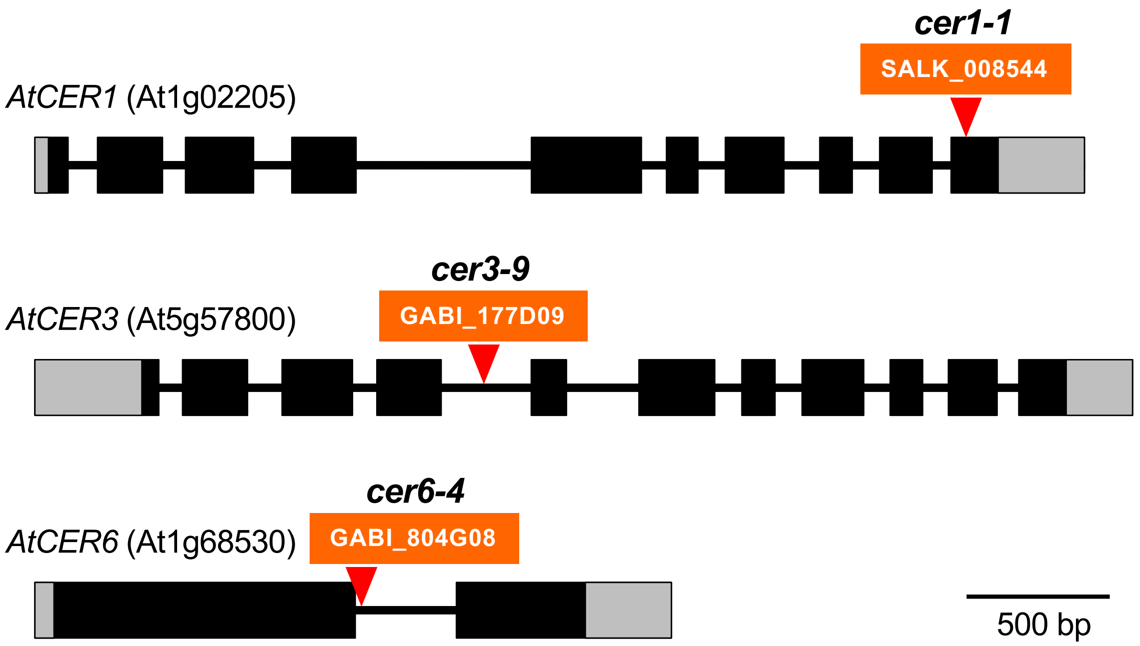

Supplementary Fig. S5
Schematic diagrams of Arabidopsis *AtCER1*, *AtCER3*, and *AtCER6* gene structures and T-DNA insertion positions of the mutant alleles used in this study. Black and gray rectangles and black lines represent the coding and noncoding exons and introns, respectively. Red triangles indicate the T-DNA positions as described in a public database.

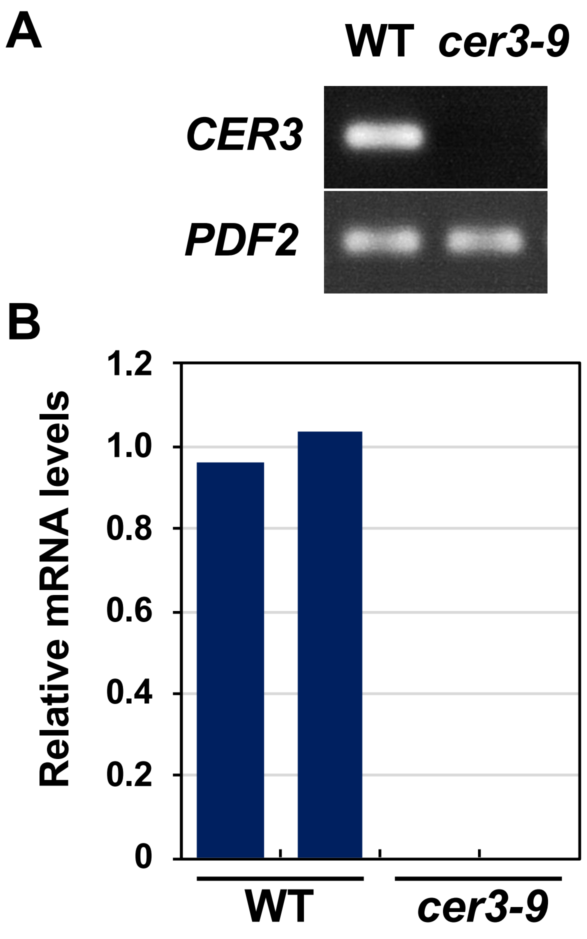

Supplementary Fig. S6
Lack of *CER3* transcripts in *cer3-9* (GABI_177D09) mutants.
(A) RNAs prepared from inflorescence stems including flower buds were analyzed by RT-PCR. In *cer3-9* mutants, no *CER3* transcripts in which exons 4 and 5 were joined were detected, indicating that the *CER3* gene in this allele was functionally disrupted by the T-DNA insertion in intron 4. *PDF2* was used as a control.
(B) Quantitative RT-PCR. Expression levels of *CER3* were normalized by *PDF2*. Two samples were measured for both WT and *cer3-9* and each measurement value is shown separately.

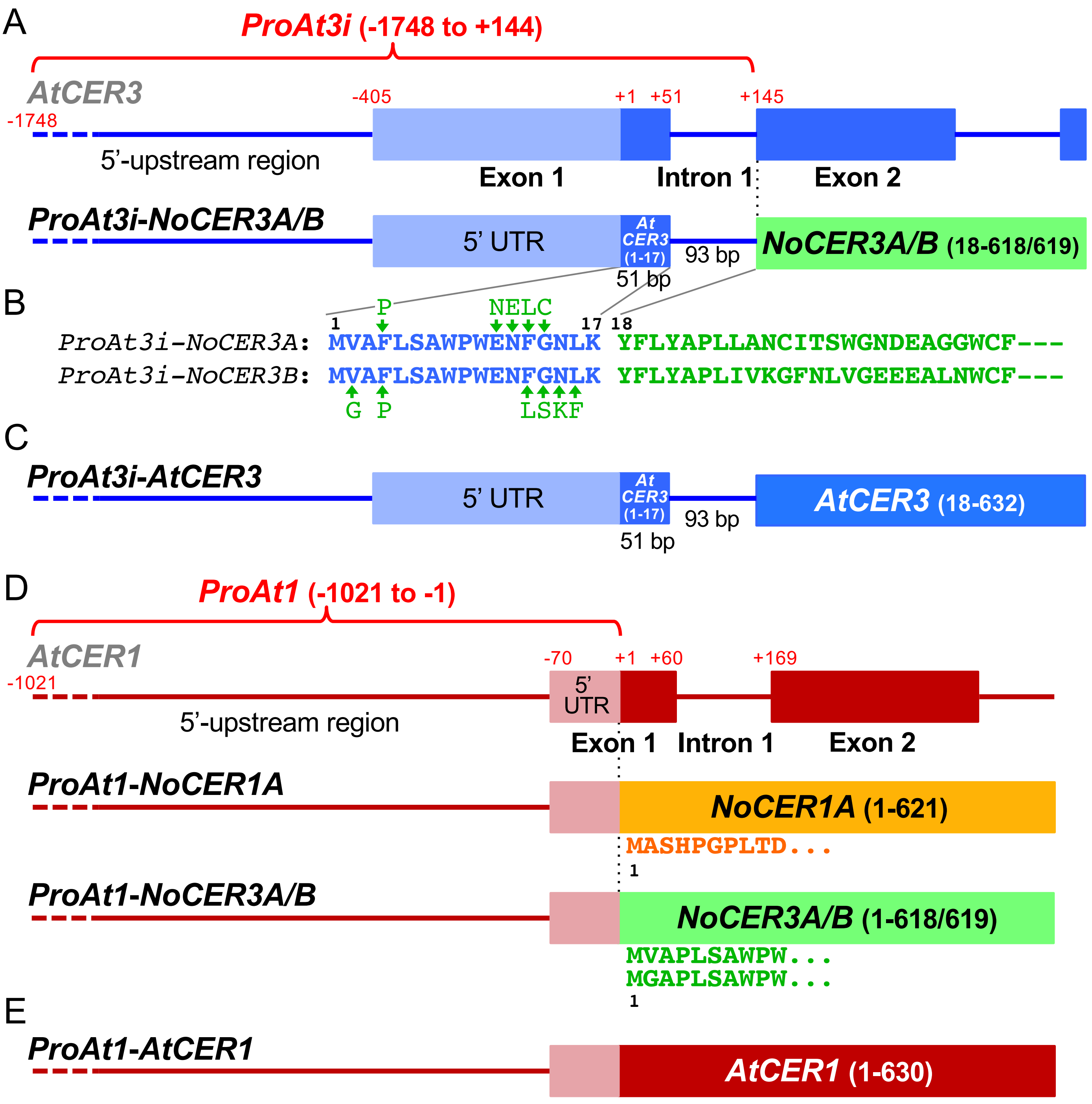

Supplementary Fig. S7

Structures of *CER1* and *CER3* genes used in the complementation experiments. **(A)** Schematic representation of N-terminal regions of the *AtCER3* gene and *ProAt3i-NoCER3A/B* fusion genes. Red numbers in *AtCER3* indicate the nucleotide position originated from A (+1) of the translational initiation codon. **(B)** Amino-acid sequences of the proteins encoded by the fusion genes. Blue and green characters represent the amino-acid residues derived from AtCER3 and NoCER3A/B, respectively. The positions of substituted residues are indicated. **(C)** Structure of the *ProAt3i-AtCER3* gene containing intron 1. **(D)** Schematic representation of N-terminal regions of the *AtCER1* gene and *ProAt1-NoCER1A* and *ProAt1-NoCER3A/B* fusion genes. Red numbers in *AtCER1* indicate the nucleotide position originated from A (+1) of the translational initiation codon. N-terminal amino-acid sequences are shown below the CDSs. **(E)** Structure of the *ProAt1-AtCER1* gene.

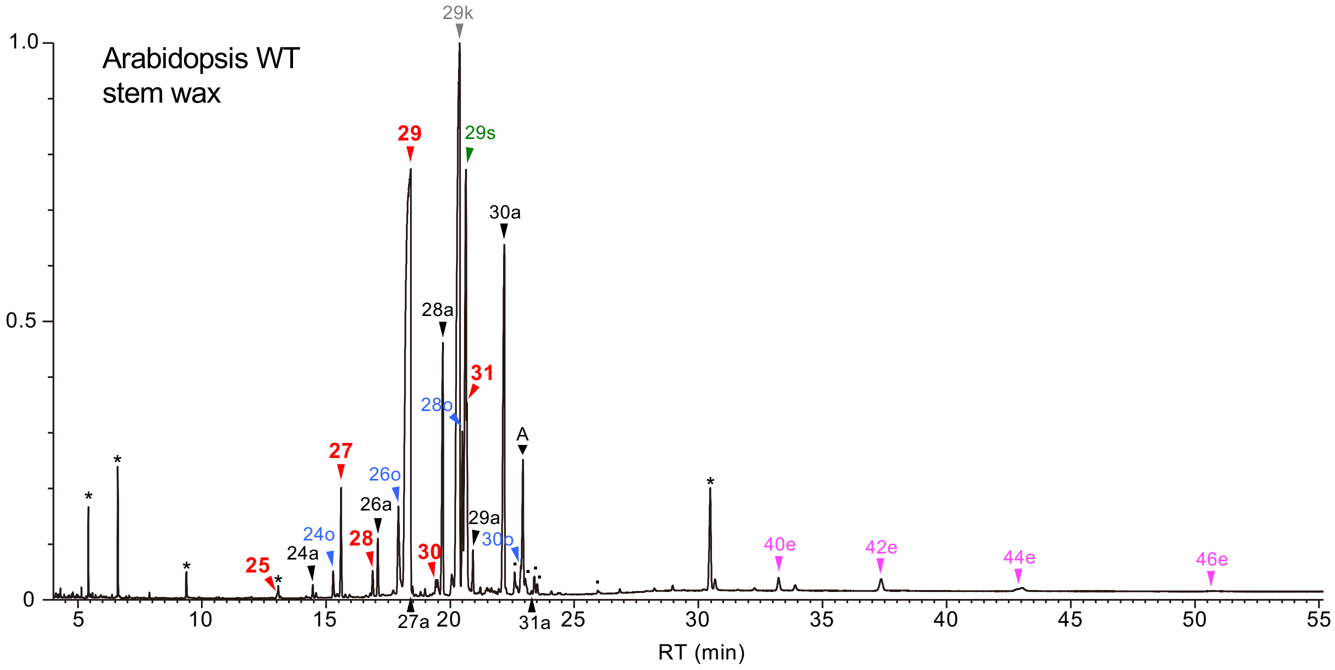

Supplementary Fig. S8

Total ion current chromatogram of an amount of stem wax of wild-type Arabidopsis analyzed by GC-MS with an extended retention time.

Magenta arrowheads with 'e' show alkyl esters of indicated carbon-chain length. Other arrowheads are explained in Figure 3 legend. Peaks with dots are sterols and terpenoids. Asterisks are artifacts due to column contamination.

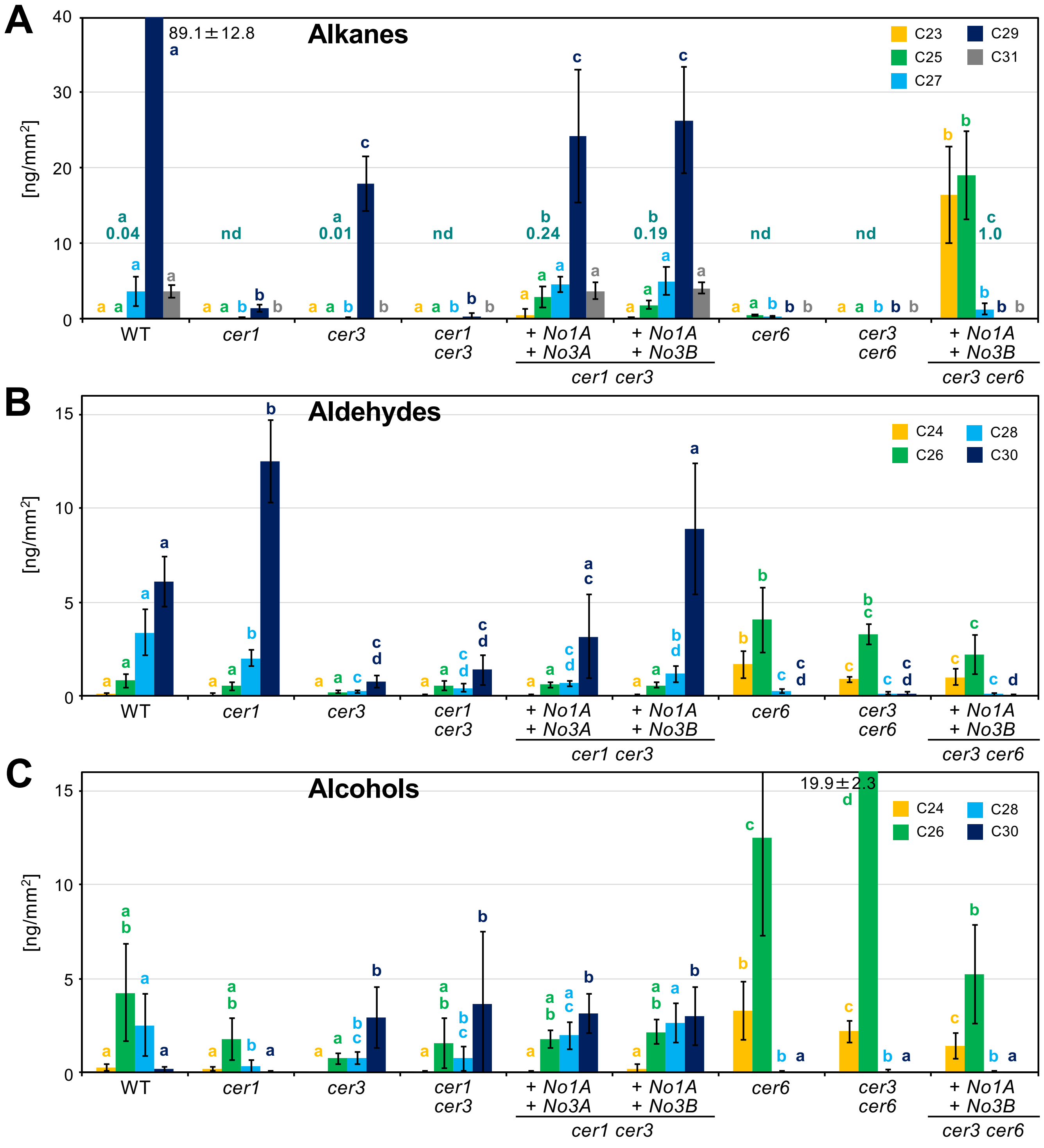

Supplementary Fig. S9

Stem cuticular wax compositions of *cer1*, *cer3*, *cer6*, their double mutants, and those expressing *NoCER1A* and *NoCER3A/B*.

Levels of odd-numbered chain length alkanes (A), even-numbered chain length aldehydes (B), and even-numbered chain length primary alcohols (C) were measured by GC-MS. The data represent means ± SD of four to eight replicates. Molecules derived from the same fatty acids are indicated by the same color. Dark green values shown in (A) represent the average ratios of C28 and shorter alkanes against total alkanes. nd, not determined due to low levels of total alkane production. Bars and values without common letters in the same color are significantly different ($P < 0.05$, Tukey-Kramer test). The analyzed transformants were T2 generation.

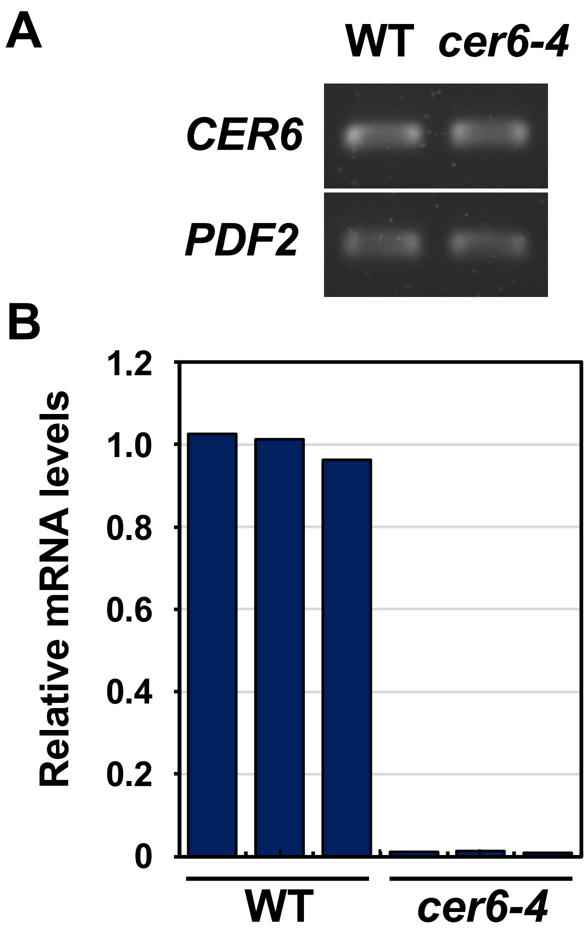

Supplementary Fig. S10
Decreased *CER6* transcripts in *cer6-4* (GABI_804G08) mutants.
(A) RNAs prepared from inflorescence stems and flower buds were analyzed by RT-PCR. A fragment in which exon 1 and exon 2 were joined were amplified indistinguishably from both WT and *cer6-4* samples by 35 cycles of PCR, suggesting that *cer6-4* was not a null allele. *PDF2* was used as a control.
(B) Quantitative RT-PCR. Expression levels of *CER6* were normalized by *PDF2*. Three samples were measured for both WT and *cer6-4* and each measurement value is shown separately.

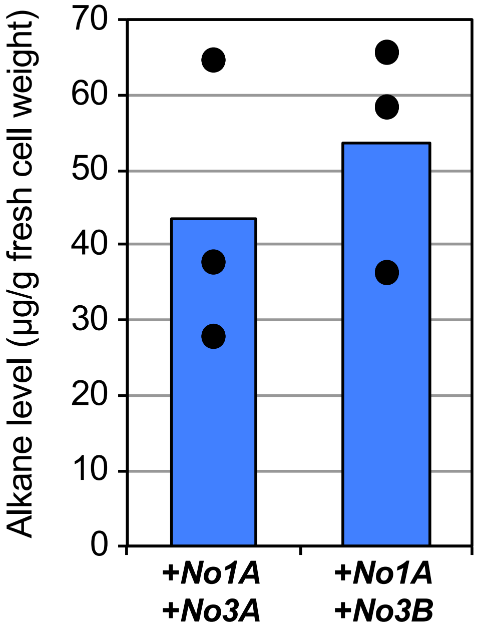

Supplementary Fig. S11
Alkane production in transgenic BY-2 cells.
Quantification of alkanes in the BY-2 calli co-expressing *NoCER1A* and *NoCER3A/B* gene pairs. A fixed amount of C15 alkane was added to the samples as an internal standard, and total alkane levels were measured by GC-MS. A dot and bar respectively represent the amount of total alkanes (C17 to C25) in an independently transformed callus and their average (n = 3). *No1A*, *Pro35S-NoCER1A*; *No3A*, *Pro35S-NoCER3A*; *No3B*, *Pro35S-NoCER3B*.

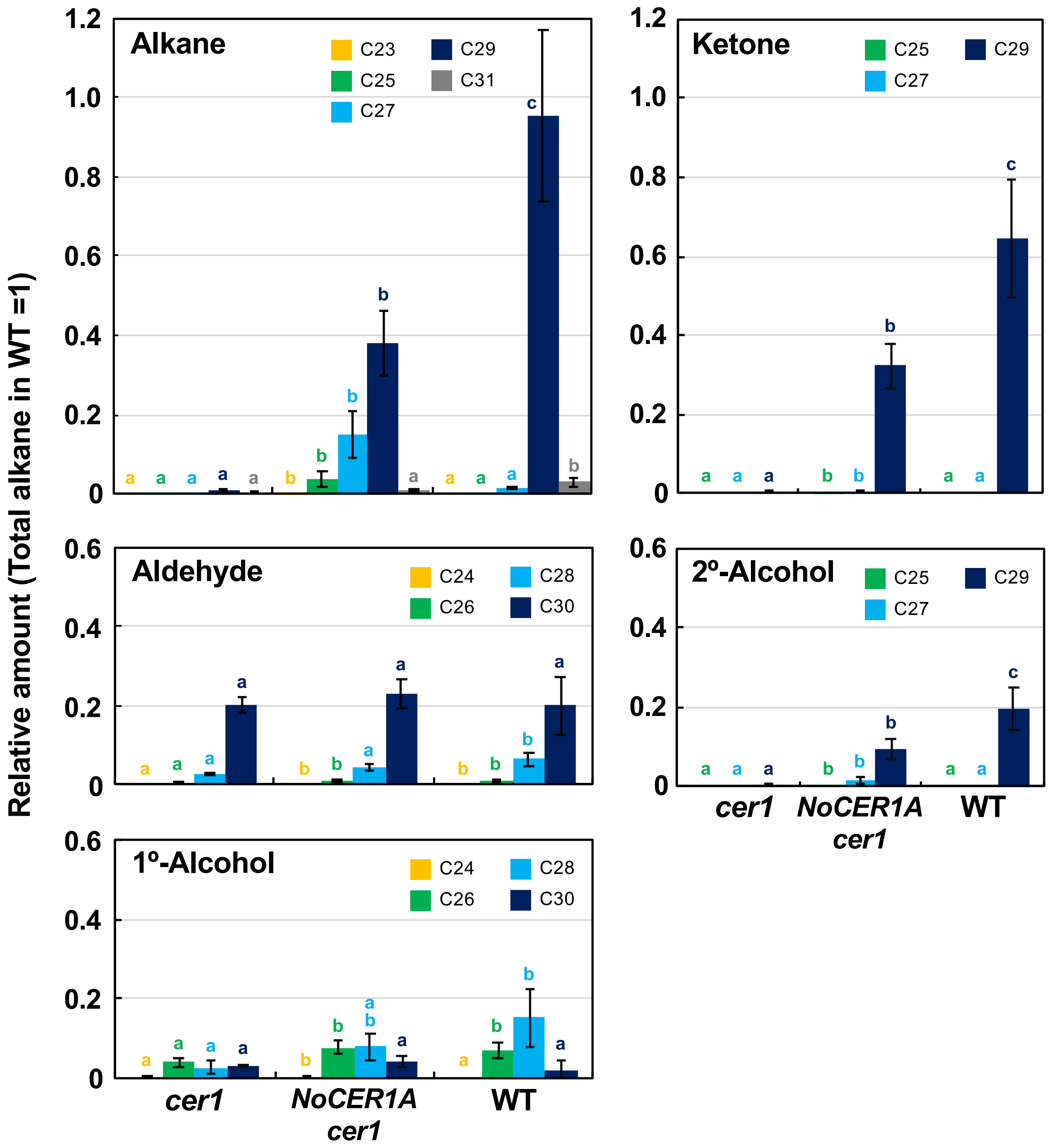

Supplementary Fig. S12

Levels of stem wax components in *cer1* mutants, those expressing *NoCER1A*, and WT plants.

Samples are the same as those used for alkane measurement in Figure 4F. The amount of each chain length species of indicated compound classes were measured by GC-MS, normalized by endogenous amyrin standard, and indicated with relative values when the total alkane level of WT is set to 1.0. Estimated relative response factors to alkane in GC-MS were 1.0 (aldehyde), 0.19 (primary alcohol), 1.0 (secondary alcohol), and 1.0 (ketone). The data represent means ± SD. Molecules derived from the same fatty acids are indicated by the same color. In each panel, bars without common letters in the same color are significantly different ($P < 0.05$, Tukey-Kramer test).

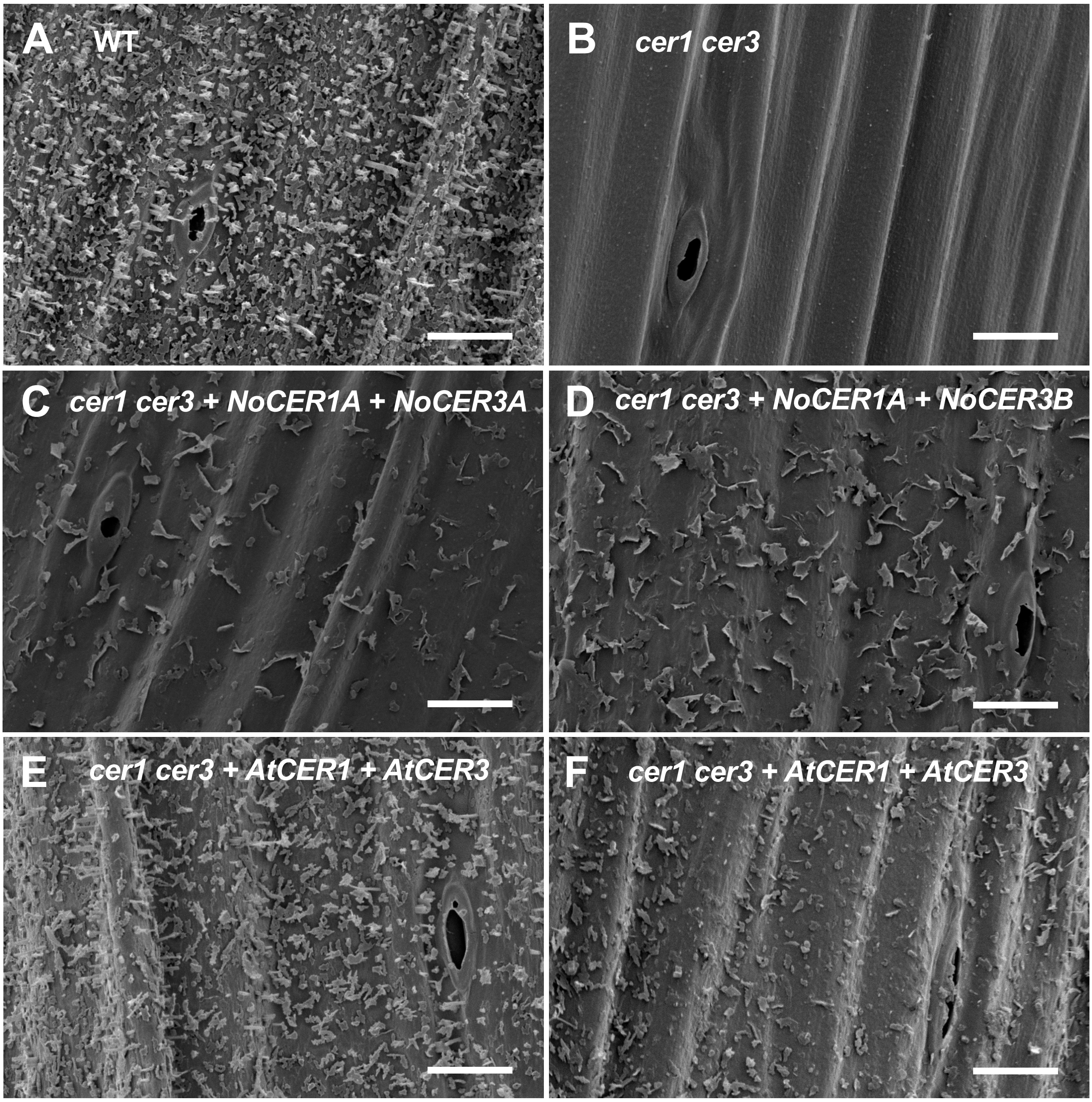

Supplementary Fig. S13
Scanning electron micrographs of the stem surface of *cer1 cer3* double mutants rescued by *CER1* and *CER3* in *N. odorata* and Arabidopsis.
(A) Wild type (control). **(B)** Untransformed cer1 *cer3* double mutant. No wax crystals were observed. **(C, D)** *cer1 cer3* plants expressing *NoCER1A* and *NoCER3A/B*. **(E, F)** *cer1 cer3* plants possessing *ProAt1-AtCER1* and *ProAt3i-AtCER3* genes. The plant in (E) produced comparable levels of alkanes to the plants shown in (C) and (D), while the plant in (F) produced less amounts of alkanes. The latter still made wax crystals rather than scales. Bar, 10 μm.

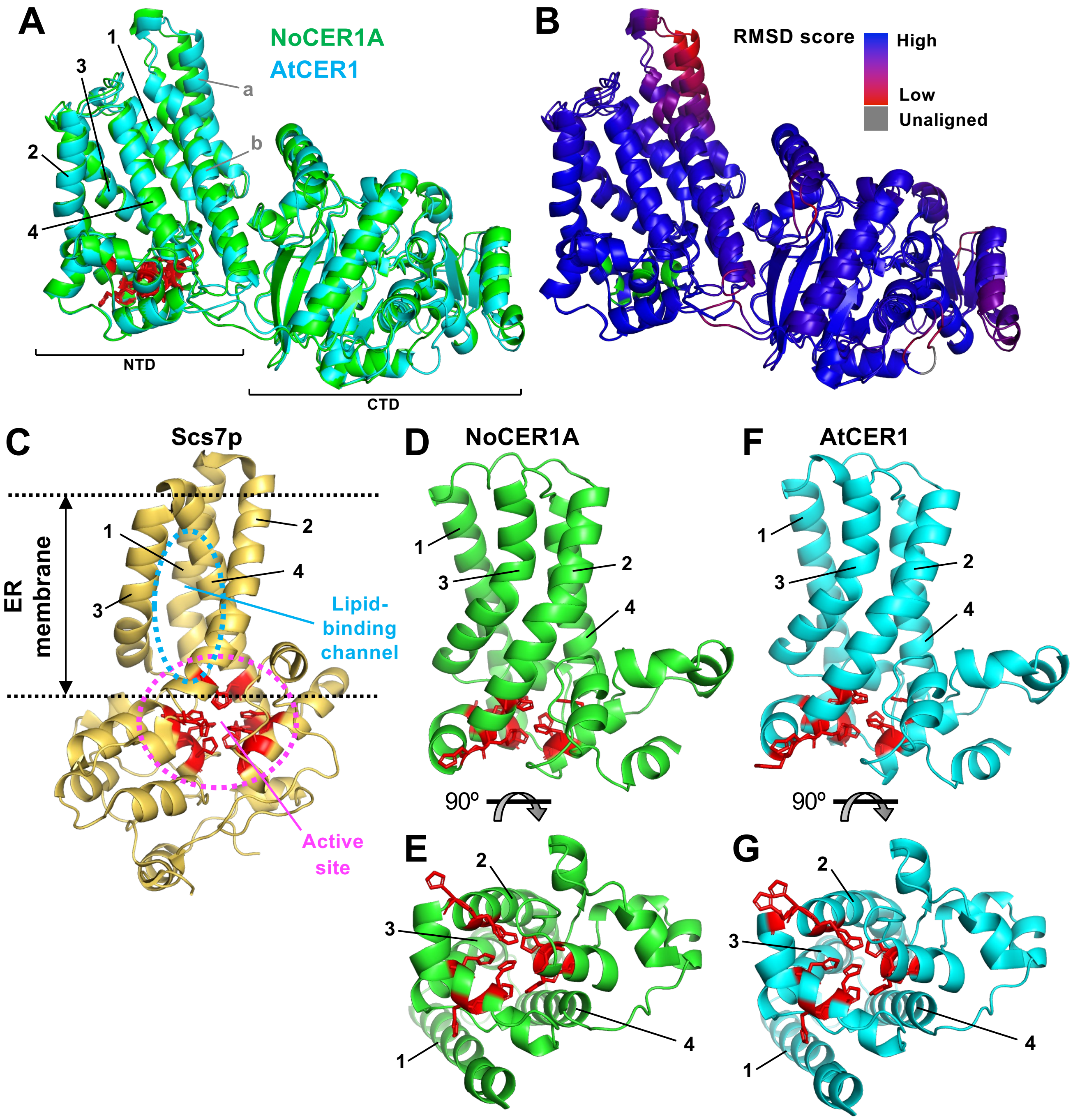

Supplementary Fig. S14

Comparison of predicted stereostructures between AtCER1 and NoCER1A.

Predicted 3D structures data served by AlphaFold Protein Structure Database was visualized by PyMOL software. **(A)** The two proteins were overlaid to compare the structures. The N-terminal and C-terminal domains (NTD and CTD) were indicated. The active site His clusters were shown in red. **(B)** The overlaid structures colored by RMSD score, which indicates the similarity between the two structures. His residues in the His clusters are green. **(C)** The crystal structure of Scs7p. **(D-G)** Side view (D and F) and bottom view (E and G) of the structures of putative lipid-binding and catalytic domains of NoCER1A (D and E) and AtCER1 (F and G), corresponding to the amino-acid residues 86 to 282. Four putative transmembrane helices forming a lipid binding cavity are numbered. Two extra transmembrane helices in NTD are labeled (a, b).

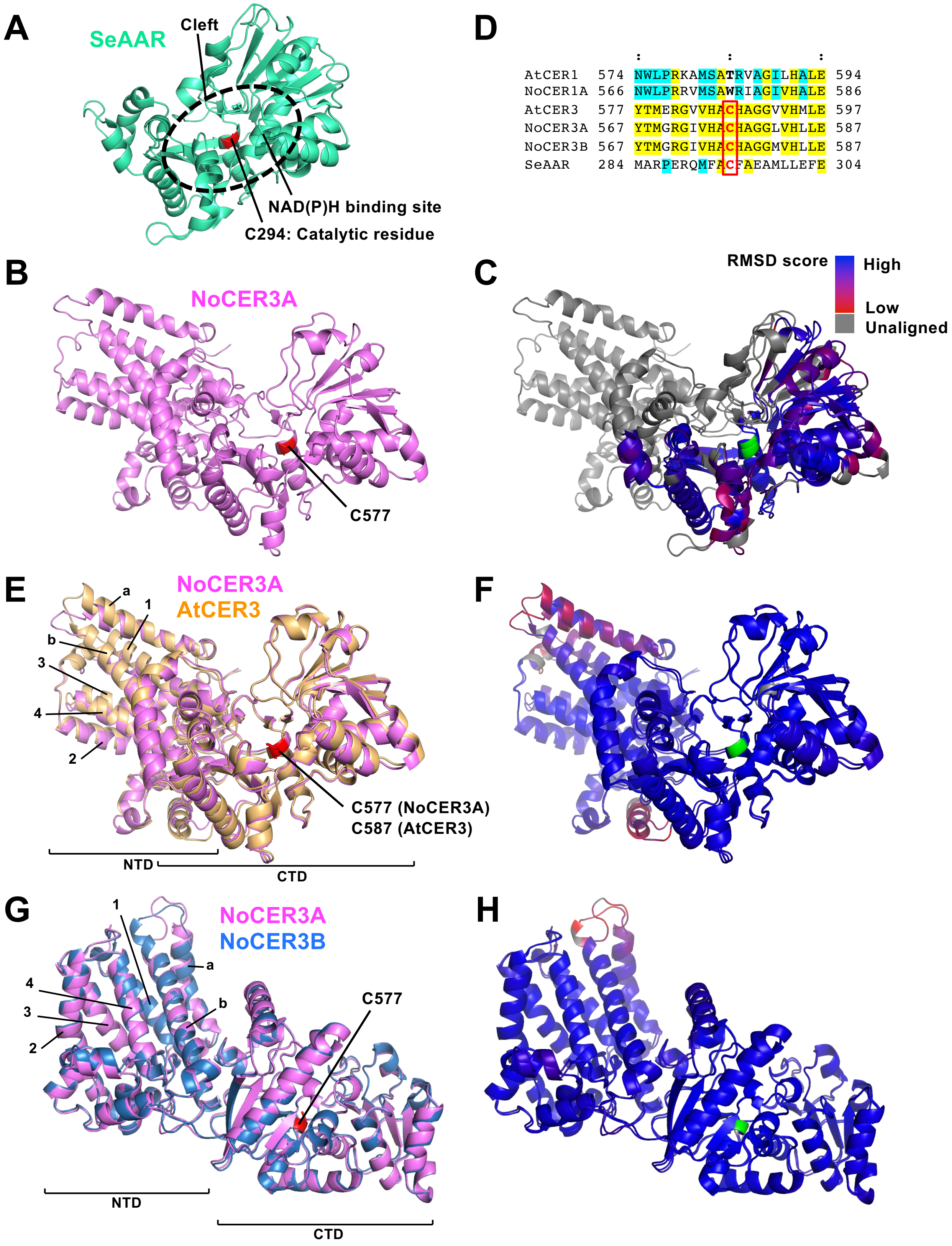

Supplementary Fig. S15

Comparison of predicted stereostructures among CER3 homologs and SeAAR.

(A) The crystal structure of SeAAR. **(B)** Alignment of the sequences around the putative catalytic cysteine residue (boxed). The residues conserved in the three CER3 homologs and those in the two CER1 homologs are painted in yellow and cyan, respectively. **(C)** A 3D structure of NoCER3A. The predicted catalytic residue, C577, is painted in red. **(D)** SeAAR and NoCER3A were overlaid and colored by RMSD score. The catalytic cysteine residue is green. **(E and F)** Comparing the structures between NoCER3A and AtCER3. Colored by RMSD score in (F). **(G and H)** Comparing the structure between NoCER3A and NoCER3B. Colored by RMSD score in (H). Note that (G) and (H) are images from a different angle to (E) and (F). Six putative transmembrane helices (a, b, and 1 to 4) are indicated.

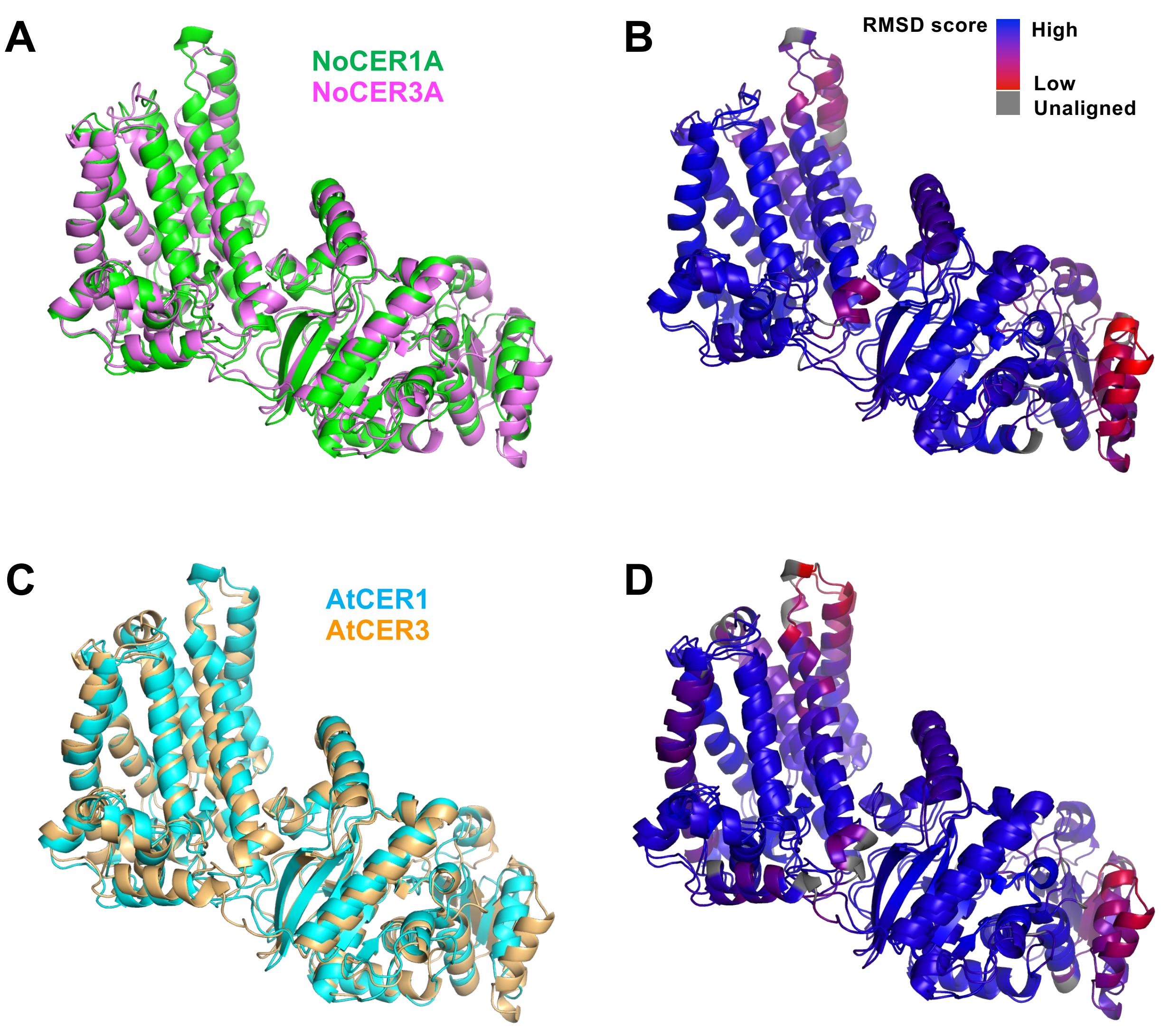

Supplementary Fig. S16
Comparison of CER1 and CER3 stereostructures.
(A and B) NoCER3A was overlaid on NoCER1A (A) and both were colored by RMSD score (B). **(C and D)** AtCER3 was overlaid on AtCER1 (C) and both were colored by RMSD score (D).

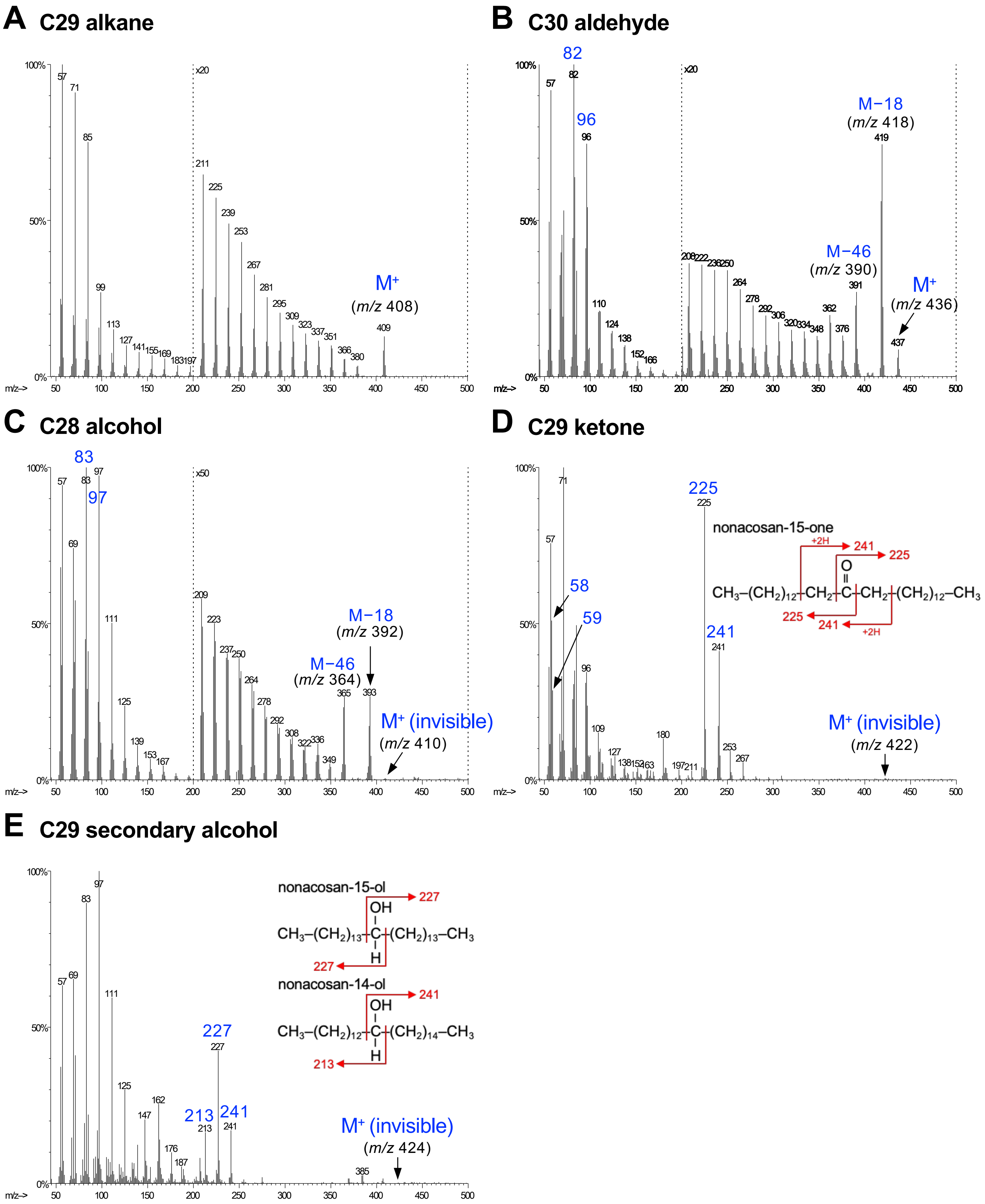

Supplementary Fig. S17

Supplementary Fig. S17

Representative mass spectrum data of each compound class in our GC-MS analysis.

(A) The C29 alkane in the wax of wild-type Arabidopsis stem. The molecular ion (M^+; *m/z* 408) peak and equally spaced peaks of fragment ions at 14 mass unit intervals are observed. Note that the molecular ion peak is observed at *m/z* 409 instead of 408 due to incomplete calibration. The peaks above *m/z* 200 are magnified by a factor of 20.

(B) The C30 aldehyde in the wax of wild-type Arabidopsis stem. The peaks of the molecular ion (*m/z* 436) and two strong [M–18] and [M–46] fragment ions, which are characteristic to long-chain fatty aldehydes, are observed (Budzikiewicz, et al., 1967; Christiansen et al., 1969; Yamamoto et al., 2008). The fragments at *m/z* 82, 96 and the following peaks separated by 14 mass unit intervals are also characteristic to the compound class (Prahl and Pinto, 1987; Yamamoto et al., 2008).

(C) The C28 alcohol in the stem wax of *cer1 cer3* double mutants. The molecular ion (m/z 410) are not detected, while the characteristic [M-18] and [M-46] fragment ions are observed (Budzikiewicz, et al., 1967; Yamamoto et al., 2008). In addition, the fragments at *m/z* 83 and 97 are commonly characteristic to alcohols in our data, though the mechanism by which these fragments are formed is not understood.

(D) The C29 ketone in the stem wax of a *cer1 cer3* plant expressing *ProAt1-NoCER1A* and *ProAt3i-NoCER3A* genes. The two fragment ions at *m/z* 225 and 241 are generated by α-cleavage and McLafferty rearrangement (β-cleavage), respectively, and the structure of this compound is therefore identified as nonacosan-15-one. The molecular ion is not visible. The fragment ions at *m/z* 58 and 59 are characteristic to ketones (Budzikiewicz, et al., 1967; Yamamoto et al., 2008).

(E) The C29 alcohol in the stem wax of *cer3*. The molecular ion is not visible, while three fragment ions at *m/z* 213, 227, and 241 are clearly observed. This data is consistent with the previous report that the C29 secondary alcohol in Arabidopsis stem wax is a mixture of nonacosan-15-ol and nonacosan-14-ol (Wen and Jetter, 2009).

Budzikiewicz, H., Djerassi, C. and Williams, D.H. (1967) Mass Spectrometry of Organic Compounds. Holden-Day, San Francisco.

Christiansen, K., Mahadevan, V., Viswanathan, R.T. and Holman, R.T (1969) Mass spectrometry of long-chain aliphatic aldehydes, dimethyl acetals and Alk-1-enyl ethers. *Lipids* 4: 421-427.

Yamamoto, S., Otto, A. and Simoneit, B.R.T (2008) GC-MS analysis of wax in leaf of *Sequoiadendron giganteum*, Sequoioideae, Cuprressace. *Res. Org. Geochem.* 23/24: 159-171.

Prahl, F.G. and Pinto, L.A. (1987) A geochemical study of long-chain n-aldehydes in Washington coastal sediments. *Geochimica et Cosmochimica Acta* 51: 1573-1582.

Wen, M. and Jetter, R. (2009) Composition of secondary alcohols, ketones, alkanediols, and ketols in *Arabidopsis thaliana* cuticular waxes. *J. Exp. Bot.* 60: 1811-1821.
