# Supplementary material for: Broad Chain-Length Specificity of the Alkane-Forming Enzymes NoCER1A and NoCER3A/B in Nymphaea odorata
Source: Plant Cell Physiol. 2024 Feb 9;65(3):428–46. doi: 10.1093/pcp/pcad168 (PMC11020225; doi:10.1093/pcp/pcad168)
Supplement: pcad168_Supp [file pcad168_supp.zip › pcad168_Supp/suppl_data/pcp-2023-e-00179-File010.pdf]

Supplemental Table S1.
Number of SNPs* in the CDSs of *N. odorata CER1* and *CER3* homologs.

Contig	CDS length	Total SNP	Nonsynonymous SNP
CER1 homolog A	1866	34 (1.8%)	10 (0.5%)
CER3 homolog A	1857	40 (2.2%)	23 (1.2%)
CER3 homolog B	1860	32 (1.7%)	13 (0.7%)

* SNPs of which the minor alleles accounted for at least 15% were counted.

Supplemental Table S2. Sequence ID of the genes shown in Fig. 2 and Supplemental Fig. S4.

Gene name	NCBI accession	Database / Gene model ID
Nymphaea odorata		
NoCER1A	LC422236	
NoCER3A	LC422237	
NoCER3B	LC422238	
Arabidopsis thaliana		TAIR (https://www.arabidopsis.org/)
AtCER1	NP_171723	At1g02205.3
AtCER1-LIKE1	NP_171721	At1g02190.1
AtCER1-LIKE2	NP_565869	At2g37700.1
AtCER3	NP_200588	At5g57800.1
Oryza sativa		RAP-DB (https://rapdb.dna.affrc.go.jp/)
OsGL1-4 (OsCER1)	Q6K9F6	Os02t0621300-01
OsGL1-5 (WDA1)	Q7XDI3	Os10t0471100-01
OsGL1-6	Q6K3D8	Os02t0814200-01
OsGL1-1 (WSL2)	Q69PA8	Os09t0426800-01
OsGL1-2	Q6ETL8	Os02t0178800-01
OsGL1-3	Q67WQ7	Os06t0653000-01
Solanum lycopersicum		Sol Genomics Network (https://solgenomics.net/)
Solyc01g088400	XP_019071187	Solyc01g088400.4.1
Solyc01g088430	XP_004229695	Solyc01g088430.4.1
Solyc03g065250	XP_004234847	Solyc03g065250.4.1
Solyc08g044260	Missing	Solyc08g044260.4.1
Solyc12g100270	XP_004252845	Solyc12g100270.2.1
Solyc03g117800	XP_010318688	Solyc03g117800.4.1
Solyc07g006300	XP_004242647	Solyc07g006300.2
Medicago truncatula		Medicago truncatula Genome Database (http://www.medicagogenome.org/)
Medtr4g054150	AES88379	Medtr4g054150.1
Medtr4g054290	XP_003606194	Medtr4g054290.1
Medtr7g090100	AES81238	Medtr7g090100.1
Medtr7g090120	XP_003625022	Medtr7g090120.1
Medtr7g090140	ABN07985	Medtr7g090140.1
Medtr8g009560	XP_003626834	Medtr8g009560.1
Medtr8g009590	XP_013443957	Medtr8g009590.1
Medtr4g129630	XP_013458551	Medtr4g129630.1
Amborella trichopoda		
AmtrCER1A	XP_006857470	
AmtrCER1B	XP_006857475	
AmtrCER3A	XP_006845484	
AmtrCER3B	XP_006836672	
Ostreocuccus tauri		Phycocosm (https://phycocosm.jgi.doe.gov/phycocosm/home)
OtCER1/3	XP_003081883.2	ostta11g00770T0
Nymphaea colorata		https://phytozome-next.jgi.doe.gov/info/Ncolorata_v1_2
Nycol.L01038	XP_031502987.1	Nycol.L01038.1
Nycol.L01040	XP_031503154.1	Nycol.L01040.1
Nycol.C01637	XP_031478996.1	Nycol.C01637.1
Nycol.I01093	XP_031496017.1	Nycol.I01093.1

Black, CER1 homologs and the common homolog in *O. tauri*; blue, CER3 homologs.

Supplemental Table S3. List of primers used in this study

	Name	Sequence
**Degenerate PCR**		
CER1	CER1+degF1	5'-ATHGAYTTYATGAAYAAYWTGGGNCAYTGCAAYTTYGA-3'
	CER1+degR1	5'-GCRCTCATNRCYYTYCTYGGNARCCARTTCTCRCA-3'
CER3	CER3+degF1	5'-AAYAAGAAYGARKCNYTNAAYGGNGG-3'
	CER3+degR1	5'-ACRTCNAKNGCNCCNACYTCRTGRTG-3'
**CDS Cloning**		
NoCER1A	EI-NyCER1	5'-CAATC*gaattc*TAAGGACAGGAGTAGCAGATCAGAGAT-3'
	NyCER1-PstI	5'-ATG*ctgcag*AACAGGCGCCTTAGGCTTATGGAAA-3'
NoCER3A	EI-NyCER3-2	5'-CCGTC*gaattc*TCTTTCTCTCTCTCTCCACAAAAAATG-3'
	NyCER3-2-PstI	5'-GGG*ctgcag*CCCTGTTTTTCATTTGATTGCAACAGGAGC-3'
NoCER3B	EI-NyCER3-1	5'-TGATC*gaattc*TTATCTCTCTCTCTCTCTGAGCACAAG-3'
	NyCER3-1-PstI	5'-CGC*ctgcag*TCTTTGAGAAGGACTACACACAACG-3'
AtCER1	CER1c+1F+attB1	5'-GGGG*acaagtttgtacaaaaaagcaggctgtata*ATGGCCACAAAACCAGGAGTCCTCA-3'
	CER1c+1890R+attB2	5'-GGGG*accactttgtacaagaaagctgggtg*ATGATGTGGAAGGAGGAGAGGCTGG-3'
AtCER3	CER3c+1F+attB1	5'-GGGG*acaagtttgtacaaaaaagcaggcttaaga*ATGGTTGCTTTTTTATCAGCTTGGCCTTGGGAAAAC-3'
	CER3c+1896R+attB2	5'-GGGG*accactttgtacaagaaagctgggtg*ATTTGTGAGTGAAGAAACAGCACTAAGACCA-3'
**Preparing recycling donor clones driven by CaMV 35S promoter**		
Pro35S	pRED419-F	5'-GTGTTCTCTCCAAATGAAATGAACTTCC-3'
	pRED419-R	5'-GAGCTCGAATTTCCCCGATCGTTC-3'
	NyCER1F-419	5'-ATTTGGAGAGAACACAAGGACAGGAGTAGCAGATCAGAG-3'
	NyCER1R-419	5'-GGGAAATTCGAGCTCGCGCCTTAGGCTTATGGAAATTACAT-3'
	NyCER3AF-419	5'-ATTTGGAGAGAACACTTTCTCTCTCTCTCTCCACAAAAAATGG-3'
	NyCER3AR-419	5'-GGGAAATTCGAGCTCCATTTGATTGCAACAGGAGCCTAGGA-3'
	NyCER3BF-419	5'-ATTTGGAGAGAACACTCTCTCTCTCTCTCTGAGCACAAG-3'
	NyCER3BR-419	5'-GGGAAATTCGAGCTCGAGAAGGACTACACACAACGTCAAGA-3'
	35S-AtCER1	5'-ATTTGGAGAGAACACCAAACATATTACATTCGACGGTATAATGG-3'
	AtCER1R-419	5'-GGGAAATTCGAGCTCAATCTTCCAAGGTTGGAGTTTTAATGATGTGGAAGGAGGAGAGGCTGG-3'
	35S-AtCER3	5'-ATTTGGAGAGAACACAAGAAGAAGAAGACCAAGCTAAAGAATG-3'
	AtCER3R-419	5'-GGGAAATTCGAGCTCAAACGTGTCTCTCTCTTCACTCAATTTGTGAGTGAAGAAACAGCACTAAGACCA-3'
**Preparing recycling donor clones driven by *AtCER1* and *AtCER3* promoters**		
ProAt-No	proAtCER1-F	5'-GGGGAC*gtcgac*GCTTTTTTCATATTCATACATCTATG-3'
	proAtCER1-R	5'-GGGGAC*gtcgac*TATACCGTCGAATGTAATATGTTTG-3'
	pRED419-pCER1-5'	5'-GAATATGAAAAAAGCGGCGTAATCATGGTCATAGCTGTTTC-3'
	pAtCER1-NyCER1	5'-ACATTCGACGGTATAATGGCTTCACACCCAGGTCCCC-3'
	proAtCER3-F	5'-GGGGAC*gtcgac*TCCCGCGTTCCCAACAAAAACGAAT-3'
	proAtCER3-R	5'-GGGGAC*gtcgac*TCTTTAGCTTGGTCTTCTTCTTCTTC-3'
	pRED419-pCER3-5'	5'-GTTGGGAACGCGGGAGGCGTAATCATGGTCATAGCTGTTTC-3'
	pAtCER3-NyCER3A	5'-AGACCAAGCTAAAGAATGGTGGCTCCACTATCTGCATGG-3'
	pAtCER3-NyCER3B	5'-AGACCAAGCTAAAGAATGGGAGCTCCACTTTCTGCGTG-3'
ProAt-At	pRED419-R	Same as above
	pAtCER1-AtCER1	5'-ACATTCGACGGTATAATGGCCACAAAACCAGGAGTCCTCA-3'
	AtCER1R-419	Same as above
	pAtCER3-AtCER3	5'-AGACCAAGCTAAAGAATGGTTGCTTTTTTATCAGCTTGGCCTTGGG-3'
	AtCER3R-419	Same as above
ProAt1-No3	pAtCER1-NyCER3A	5'-ACATTCGACGGTATAATGGTGGCTCCACTATCTGCATGG-3'
	NyCER3AR-419	Same as above
	pAtCER1-NyCER3B	5'-ACATTCGACGGTATAATGGGAGCTCCACTTTCTGCGTG-3'
	NyCER3BR-419	Same as above
ProAt3i-No3	AtCER3in-Ny3A-2eR	5'-TGCATATAAAAAATACTTCACATATAGACAACAAAATCA-3'
	AtCER3in-Ny3B-2eR	5'-TGCATATAAGAAATACTTCACATATAGACAACAAAATCA-3'
	NyCER3A-2nd-ex	5'-TATTTTTTATATGCACCGCTTCTAGC-3'
	NyCER3B-2nd-ex	5'-TATTTCTTATATGCACCCTTGATAG-3'
ProAt3i-At3	AtCER3in-At3-2eR	5'-AGCGTAGAGAAGATACTTCACATATAGACAACAAAATCAC-3'
	AtCER3-2nd-ex	5'-TATCTTCTCTACGCTCCATTAGCTGC-3'
**RT-qPCR for evaluating Arabidopsis *cer3* and *cer6* mutants**		
AtCER3	CER3+918F	5'-TAAGTGCGATCTACGGTTACGCTGT-3'
	CER3+1394R	5'-GGTCCCCATTTCTTGATGATGCAGA-3'
CER6	CER6+1017F	5'-AGCCCTCAAGGCAAACATCACCACA-3'
	CER6+1158R	5'-GAAGGCCAGCTTGAAATCCGGTATG-3'
PDF2	PDF2cds+934F	5'-AACCCTGAACTCGCTATCCAGCACA-3'
	PDF2cds+1065R	5'-TGTTGCATCCTTACCCAAGACTGGA-3'
