# Supplementary material for: Broad Chain-Length Specificity of the Alkane-Forming Enzymes NoCER1A and NoCER3A/B in Nymphaea odorata
Source: Plant Cell Physiol. 2024 Feb 9;65(3):428–46. doi: 10.1093/pcp/pcad168 (PMC11020225; doi:10.1093/pcp/pcad168)
Supplement: pcad168_Supp [file pcad168_supp.zip › pcad168_Supp/suppl_data/pcp-2023-e-00179-File012.pdf]

Supplemental Methods.

Construction of plasmids for *CER1* and *CER3* expression.

All the primers used for plasmid construction are listed in Supplementary Table S3. Note that the names NyCER1A, NyCER3A, and NyCER3B, which are previous gene names of NoCER1A, NoCER3A, and NoCER3B, respectively, remain in the names of plasmids and primers.

Recycling donor clones for *Pro35S*-driven *CER1/3* genes

The Gateway recycling cloning system was applied for the construction of all plant transformation plasmids used in this study (Kimura et al., 2013). The starting plasmid for the construction was pRED419-Amp-P_{35S}:GUS:Tnos, in which an unique *Hin*d III-*Eco*R I fragment of pBI221 (Clontech) was subcloned between the *Hin*d III and *Eco*R I sites of pRED419-Amp (http://shimane-u.org/nakagawa/gbv.htm), a derivative of pRED419 (Kimura et al., 2013). The *Hin*d III-*Eco*R I fragment contained *Pro35S*, the β-glucuronidase gene (GUS), and a *nos* terminator (Tnos). To remove the GUS gene from the plasmid, PCR was carried out with primers pRED419-F and pRED419-R using PrimeSTAR HS DNA polymerase (Takara Bio). The CDS fragment of *NoCER1A* was prepared by PCR using a pUC-NyCER1A template and NyCER1F-419 and NyCER1R-419 primers. The two fragments were connected by In-Fusion cloning kit (Clontech), and the resulting plasmid was named pRED-35SpNyCER1A.

The CDS fragments of *NoCER3A*, *NoCER3B*, *AtCER1*, and *AtCER3* were similarly prepared by PCR with primers listed in Table S2 and were connected with the above vector fragment containing *Pro35S*. The resulting plasmids were pRED-35SpNyCER3A, pRED-35SpNyCER3B, pRED-35SpAtCER1, and pRED-35SpAtCER3.

Recycling donor clones for *ProAt1-NoCER1A* and *ProAt3-NoCER3A/B* genes

To prepare the *ProAt1-NoCER1A* cassette, a DNA fragment spanning the *AtCER1* promoter (–1021 to –1) was prepared by PCR amplification from Arabidopsis Col-0 genomic DNA using primers proAtCER1-F and proAtCER1-R and subsequent *Sal* I digestion. Another PCR was carried out with a pRED-35SpNyCER1A plasmid and pRED419-pCER1-5’ and pAtCER1-NyCER1 primers to prepare a promoter-removed vector/CDS fragment. Two fragments were connected by In-Fusion cloning kit to produce pRED-1pNyCER1A. For preparation of *ProAt3-NoCER3A/B* cassettes, a fragment of the *AtCER3* promoter (–1748 to –1) was prepared by PCR using proAtCER3-F and proAtCER3-R primers with subsequent

Sal I digestion. Vector/CDS fragments containing NoCER3A and NoCER3B were similarly prepared from pRED-35SpNyCER3A and pRED-35SpNyCER3B plasmids using pRED419-pCER3-5’ and pAtCER3-NyCER3A (or pAtCER3-NyCER3B) primers. The resulting plasmids were pRED-3pNyCER3A and pRED-3pNyCER3B.

Recycling donor clones for *ProAt1-AtCER1* and *ProAt3-AtCER3* genes

The *NoCER1A* CDS in pRED-1pNyCER1A was replaced by *AtCER1* as follows. A vector/promoter fragment was prepared from the plasmid by PCR-amplification using proAtCER1-R and pRED419-R primers. The *AtCER1* CDS fragment was prepared by PCR from pDONR-AtCER1 using pAtCER1-AtCER1 and AtCER1R-419 primers. The two fragments were connected by In-Fusion cloning kit to make pRED-1pAtCER1. pRED-3pAtCER3 was prepared similarly from pRED-3pNyCER3A and pDONR-AtCER3 using proAtCER3-R and pRED419-R primers for vector/promoter amplification and pAtCER3-AtCER3 and AtCER3R-419 primers for CDS amplification.

Recycling donor clones for *ProAt1-NoCER3A/B* genes

PCR-amplification of NoCER3A CDS and NoCER3B CDS was carried out by use of pRED-3pNyCER3A (or pRED-3pNyCER3B) plasmid with pAtCER1-NyCER3A and NyCER3AR-419 (or pAtCER1-NyCER3B and NyCER3BR-419) primers. The CDS fragments were connected with the above described vector/promoter fragment prepared from pRED-1pNyCER1 to form pRED-1pNyCER3A and pRED-1pNyCER3B.

Recycling donor clones for *ProAt3i-NoCER3A/B* genes

A DNA fragment spanning the AtCER3 promoter, its first exon, and its first intron was prepared by PCR-amplification from Arabidopsis Col-0 genomic DNA. The primers were AtCER3in-Ny3A-2eR and proAtCER3-F for NoCER3A, and the former was replaced by AtCER3in-Ny3B-2eR for NoCER3B. The CDS fragments of NoCER3A and NoCER3B genes lacking an N-terminal 51-bp region were PCR-amplified from pRED-3pNyCER3A (or pRED-3pNyCER3B) using NyCER3A-2nd-ex (or NyCER3B-2nd-ex) primer and a common pRED419-pCER3-5’ primer. The promoter and CDS (with vector) fragments were connected by In-Fusion cloning kit, and the resulting plasmids were named pRED-3ipNyCER3A and pRED3ipNyCER3B.

Recycling donor clone for *ProAt3i-AtCER3* gene

A DNA fragment spanning the AtCER3 promoter, its first exon, and its first intron was

prepared by PCR-amplification from pRED-3ipNyCER3A with two primers AtCER3in-At3-2eR and pRED419-R. The CDS fragment of AtCER3 lacking its first exon was PCR-amplified from pRED-3pAtCER3 using AtCER3-2nd-ex and AtCER3R-419 primers. The promoter and CDS (with vector) fragments were connected by In-Fusion cloning kit, and the resulting plasmid was named pRED-3ipAtCER3.

Construction of binary plasmids harboring *CER1* and *CER3* expression cassettes

To construct the binary plasmid harboring a single expression cassette for one of *NoCER1A*, *NoCER3A*, or *NoCER3B*, an appropriated recycling donor clone was reacted with a destination vector, pGWB501, by Gateway LR reaction (Thermo-Fisher Scientific). To construct the binary plasmid harboring both *CER1* and *CER3* expression cassettes, a recycling donor clone carrying *CER3* was first reacted with pGWB501 by Gateway LR reaction. Then, the resulting plasmid was reacted with a conversion vector pCON-Cm-rare2 (a derivative of pCON; http://shimane-u.org/nakagawa/gbv.htm) by Gateway LR reaction (Kimura et al., 2013). This plasmid was reacted with a second recycling donor clone carrying the *CER1* expression cassette. The finally obtained plasmid contained *CER3* and *CER1* expression cassettes in this order.

Evaluation of novel *cer3* and *cer6* alleles

Flower bud clusters and stems up to 40 mm from the apex were harvested. After all open flowers and young fruits were removed, remaining bud clusters, stems, and pedicels were used for RNA preparation using RNeasy Mini Kit (Qiagen). Reverse transcription was performed with ReverTra Ace qPCR RT Master Mix with gDNA Remover (Toyobo). PCR was carried out with Quick Taq HS Dye mix (Toyobo). Quantitative PCR was performed using Thunderbird SYBR qPCR Mix (Toyobo) and StepOne Realtime PCR System (Applied Biosystems). *PDF2* (PP2A A3; At1g13320) was used as a standard for quantification. Primers used for PCR and qPCR were listed in Supplementary Table S3.

REFERENCE

Kimura, T., Nakao, A., Murata, S., Kobayashi, Y., Tanaka, Y., Shibahara, K., Kawazu, T. and Nakagawa, T. (2013) Development of the gateway recycling cloning system for

multiple linking of expression cassettes in a defined order, and direction on gateway compatible binary vectors. *Biosci. Biotechnol. Biochem.*, **77**, 430-434.
